# Course of Chronic *Trypanosoma cruzi* Infection after Treatment Based on Parasitological and Serological Tests: A Systematic Review of Follow-Up Studies

**DOI:** 10.1371/journal.pone.0139363

**Published:** 2015-10-05

**Authors:** Yanina Sguassero, Cristina B. Cuesta, Karen N. Roberts, Elizabeth Hicks, Daniel Comandé, Agustín Ciapponi, Sergio Sosa-Estani

**Affiliations:** 1 Centro Rosarino de Estudios Perinatales (CREP), Cochrane Centre CREP, Rosario, Santa Fe, Argentina; 2 Instituto Nacional de Parasitología (INP), “Dr Mario Fatala Chaben”, Administración Nacional de Laboratorios e Institutos de Salud (ANLIS) Malbrán, Buenos Aires, Argentina; 3 Facultad de Ciencias Económicas y Estadística, Universidad Nacional de Rosario, Santa Fe, Argentina; 4 Duke University School of Medicine, Durham, North Carolina, United States of America; 5 Instituto de Efectividad Clínica y Sanitaria (IECS), Cochrane Centre IECS, Buenos Aires, Argentina; University of Sao Paulo, BRAZIL

## Abstract

**Background:**

Chagas disease is caused by the flagellate protozoan Trypanosoma cruzi (*T*. *cruzi*). It is endemic in Latin American countries outside the Caribbean. The current criterion for cure in the chronic phase of the disease is the negativization of at least two serological tests such as enzyme-linked immunosorbent assay (ELISA), indirect immunofluorescence assay (IIF) and indirect hemagglutination assay (IHA). The serological evolution of treated subjects with chronic *T*. *cruzi* infection is variable. Treatment failure is indicated by a positive parasitological and/or molecular test (persistence of parasitemia).

**Objectives:**

To summarize the pattern of response to treatment of parasitological, molecular and serological tests performed during the follow-up of subjects with chronic *T*. *cruzi* infection.

**Methods:**

Electronic searches in relevant databases and screening of citations of potentially eligible articles were accomplished. Organizations focusing on neglected infectious diseases were asked for help in identifying relevant studies.

Included studies were randomized controlled trials (RCTs), quasi-RCTs, and cohort studies involving adults and children with chronic infection who received trypanocidal treatment (benznidazole or nifurtimox) and were followed over time. The assessment of risk of bias was performed separately for each study design. The Cochrane Collaboration’s tool and the guidelines developed by Hayden et al. were used. Two reviewers extracted all data independently. A third review author was consulted in case of discordant opinion.

Additional analyses were defined in ad-hoc basis. Scatter plots for percentage of positive parasitological and molecular tests and for negative serological tests were developed by using the lowess curve technique. Heterogeneity was measured by I^2^.

The protocol was registered in PROSPERO, an international prospective register of systematic review protocols (Registration Number CRD42012002162).

**Results:**

Out of 2,136 citations screened, 54 studies (six RCTs and 48 cohort studies) were included. The smoothed curves for positive xenodiagnosis and positive polymerase chain reaction (PCR) were characterized by a sharp decrease at twelve month posttreatment. Afterwards, they reached 10–20% and 40% for xenodiagnosis and PCR, respectively. The smoothed curves for negative conventional serological tests increased up to 10% after 48 months of treatment. In the long-term, the rate of negativization was between 20% and 45%.

The main sources of bias identified across cohort studies were the lack of control for confounding and attrition bias. In general, RCTs were judged as low risk of bias in all domains. The level of heterogeneity across included studies was moderate to high.

Additional analysis were incomplete because of the limited availability of data. In this regard, the country of origin of study participants might affect the results of parasitological and molecular tests, while the level of risk of bias might affect serological outcomes. Subgroup analysis suggested that seronegativization occurs earlier in children compared to adults.

**Conclusions:**

We acknowledge that there is a dynamic pattern of response based on parasitological, molecular and serological tests in subjects chronically infected with *T*. *cruzi* after treatment. Our findings suggest a trypanocidal effect in the long-term follow-up.

Further research is needed to explore potential sources of heterogeneity and to conduct reliable subgroup analysis.

## Introduction

Chagas disease is caused by the flagellate protozoan *Trypanosoma cruzi* (*T*. *cruzi*). It is endemic in 21 countries in the Americas, with more cases appearing globally as infected individuals migrate. Chagas disease has expanded from rural to urban areas and endemic to non-endemic regions [1]. It has been estimated that 10 million people are chronically infected with *T*. *cruzi* [2,3] and that 12,000 people die each year as a result [4]. Moreover, 25 million people are at risk of having the disease [3].

The disease is principally transmitted through vectors, namely triatomine bugs. The parasite may also be transmitted through blood transfusions, congenital transmission from an infected mother to child, organ donation, laboratory accidents, needle sharing among injecting drug users, or orally through food and drink contaminated with insects or feces with parasites [1]. The disease evolves in two phases, acute and chronic, each of them with different clinical characteristics and diagnostic criteria.

Timely treatment with trypanocidal drugs (benznidazole and nifurtimox) is effective to cure acute *T*. *cruzi* infection [5].

Subjects who do not receive etiological treatment during the acute phase go on to develop chronic infection, which begins when the presence of the parasite in the blood becomes undetectable through parasitological or molecular tests. An absence of clinical symptoms or signs of visceral lesions characterize it. The indeterminate form (i.e., “phase without demonstrated pathology”) may persist for the lifetime of the patient or it may progress into the chronic phase with a cardiac, digestive, neurological, or mixed form after fifteen to twenty years. Long-term complications of chronic *T*. *cruzi* infection include dysrhythmias, heart failure, megaesophageus and megacolon.

The diagnosis of infection in the chronic phase is done by serological techniques that screen for IgG antibodies against internal or surface components of intact *T*. *cruzi* or its extracts [5]. Three conventional assays are widely used: enzyme-linked immunosorbent assay (ELISA), the indirect immunofluorescence assay (IIF) and the indirect hemagglutination assay (IHA). At present, the use of at least two different assays to confirm diagnosis of the disease is recommended.

There are also certain non-conventional tests that are based on ELISA techniques and use reagents such as recombinant proteins, purified antigens, or synthetic peptides. Some of these tests are already available on the market, but the majority can be obtained only from their producers. If the results of the two classes of tests are not in agreement, it is advised that those of the conventional serology should be taken as truthful.

Although specific, xenodiagnosis has low sensitivity (20–50%) during chronic phase compared to PCR (30–80%) [6].

Trypanocidal therapy given to children or adults with chronic *T*. *cruzi* infection is associated with a beneficial effect on parasite-related outcomes [7–9]. It is agreed that negative parasitological tests do not ensure the absence of infection while positive parasitological results are useful to determine treatment failure.

The current criterion for cure of chronic *T*. *cruzi* infection consists of two non-reactive conventional serological assays [5] using crude parasite antigens and/or recombinant antigens which are available in endemic countries as commercially diagnostic kits. The variability in the serological evolution of treated subjects [10,11] may be explained by the host capability to control the infection through the immune system.

The probability of cure might be also predicted by a decreasing antibody titres for *T*. *cruzi* over time.

Among the variables that affect the post-therapeutic response in the chronic phase, we highlight: the time elapsed since the infection was acquired (a parameter difficult to determine), the age at treatment, the time between treatment and follow-up, the laboratory method used to assess response to treatment, and the region where the subject was infected [12]. In this scenario, it is admitted that: i.-antibody titres decline more slowly in adults than in children, ii.-subjects who were closer to the acute phase of the disease at the time of treatment may seroconvert more rapidly, and iii.-studies with longer follow-up show higher rates of serological negativization [13].

### Main Results from Existing Systematic Reviews on the Topic

Four systematic reviews assessing the effectiveness of treatment and reporting on parasite-related outcomes were identified [7–9,14]. The review published by Villar et al. [7] assessed seropositive individuals to *T*. *cruzi* who received any treatment versus placebo or no treatment. Results for positive xenodiagnosis showed an odds ratio (OR) of 0.35 (95% confidence interval (CI) 0.14 to 0.86; six studies; I^2^ 79%). The OR for positive PCR was 0.50 (95% CI 0.27 to 0.90; two studies; I^2^ 0%). Treatment for chronic *T*. *cruzi* infection was also associated with heterogeneous reductions in the chance of persistence of positive serology (OR = 0.21, 95% CI 0.10 to 0.44; I^2^ 76%), although it included one RCT and one observational study testing allopurinol in adults.

The review published by Vallejo [14] included one RCT involving patients with chronic chagasic cardiopathy with a minimum follow-up of 18 months.

The review published by Rodrigo Fuentes et al. [8] included subjects with chronic asymptomatic Chagas disease treated with nifurtimox. The main outcome measure was the natural logarithm of the chance of negativization of serological and parasitological tests in the absence of clinical signs (log odds = 0.37, 95% CI 1.32 to 2.07; seven studies).

The review published by Pérez-Molina et al. [9] included chronically infected patients who were treated with benznidazole compared with no treatment or placebo. Parasitological and serological outcomes were combined into one single meta-analysis showing that benznidazole increases by18-fold the probability of a beneficial response (OR = 18.8, 95% CI 5.2 to 68.3; nine studies; I^2^ 76%).

In the light of the evidence derived from published meta-analyses, our systematic review is relevant for the purpose of answering a still open question: when and to what extent the administration of currently recommended trypanocidal drugs carries the negativization of serology tests used to monitor subjects chronically infected. Our hypothesis is that the rate of seronegativization after treatment is about 60–70% in children who have been treated before fifteen years of age, and 30–50% in adults after fifteen to twenty years of follow-up.

Our main objective is to summarize the patterns of response to treatment of parasitological, molecular and serological tests performed during the short, medium and long-term follow-up of subjects with chronic *T*. *cruzi* infection.

## Materials and Methods

### Eligibility Criteria

Eligible studies were randomized, quasi-randomized trials (i.e., allocation of treatment done on the basis of a pseudo-random sequence such as odd/even hospital number or date of birth), and cohort studies including adults and children with chronic *T*. *cruzi* infection who received trypanocidal treatment and were followed over time. Chronic Chagas disease was defined as *T*. *cruzi* infection confirmed with at least two positive serological tests. Benznidazole 5-7mg/kg per day or nifurtimox 10-12mg/kg (infants) or 8-10mg/kg (adults) for 30–60 days were considered as adequate treatment. No limit in the duration of follow-up was applied.

We excluded studies involving subjects in the acute phase when they received treatment; subjects treated with itraconazole or allopurinol; children aged twelve months or younger born to infected mothers; and immunocompromised patients or pregnant women.

The laboratory tests considered were parasitological tests such as hemoculture, xenodiagnosis or PCR, and serological assays such as ELISA, IIF, IHA, complement fixation (CF), direct hemagglutination with and without 2-mercaptoethanol (DA-2ME) or complement mediated lysis (CoML). We included other tests not commercialized, also called non-conventional, ELISA tests as reported by researchers.

Our outcomes of interest were positive parasitological and molecular tests (persistence of parasitemia) and negative serological tests (disappearance of specific *T*. *cruzi* antibodies). We were not able to include other outcomes such as decrease of *T*. *cruzi* antibodies level because of reporting pitfalls.

### Search Strategy

The following databases were searched on July 28^th^ 2013: the Cochrane Database of Systematic Reviews (CDSR), Cochrane Central Register of Controlled Trials (CENTRAL), Database of Abstracts of Reviews of Effects (DARE), MEDLINE, EMBASE, and LILACS.

The search strategy included the following terms: (Chagas Disease[Mesh] OR Chagas[tiab] OR Trypanosom*[tiab] OR Cruzi[tiab] OR T.Cruzi[tiab]) AND (Benzonidazole[Supplementary Concept] OR benznidazol*[tiab] OR Radanil[tiab] OR Nifurtimox[Mesh] OR nifurtimox[tiab] OR Lampit[tiab]).

The search terms were modified to suit the requirements of particular databases, where necessary. No language limitations or publication date restrictions were applied.

We explored the following Trials Registers on December 2^nd^ 2013: ClinicalTrials.gov (http://www.clinicaltrials.gov/), MetaRegister of Controlled Trials (http://www.controlled-trials.com/mrct/), and WHO International Clinical Trials Registry ((http://apps.who.int/trialsearch/).

The reference lists of retrieved articles and relevant reviews were checked for potentially eligible studies [15]. Researchers from organizations focusing on neglected infectious diseases were contacted by email asking for help in identifying potentially relevant studies that could be missed through our electronic searches.

The following organizations were approached: The World Health Organization (www.who.int/tdr), The Pan-American Health Organization (www.paho.org/chagas), The UK Department for International Development (http://r4d.dfid.gov.uk/SiteSearch.aspx?q=chagas), Fundación Mundo Sano (www.mundosano.org), American Society of Tropical Medicine and Hygiene (http://www.astmh.org), and Fiocruz (http://portal.fiocruz.br).

### Study Selection

Three review authors independently screened titles and abstracts of articles through the web-based software Early Review Organizing Software [16], one assessed all articles and each of the other reviewers assessed half of them. All studies potentially eligible for inclusion were reviewed independently in full-text format by the principal investigators (YS and SSE).

### Data Extraction

Two reviewers (YS and EH) independently extracted and recorded general data from each included study. In case of discordant opinion, a third review author (SSE) was consulted for consensus. A pre-designed general data extraction form was used. It included the following information:

- Source of study report: publication type, year of publication, journal, and authors’ names.
- Study location: geographical region, country and province/city.
- Study population: sample size, age at enrollment, living in endemic area under or not under surveillance, visit to endemic area (even for a brief period) and dates of initiation and ending of data collection.
- Disease: definition of chronic *T*. *cruzi* infection, diagnostic tests (number and type of laboratory tests used) and internal quality control measures.
- Trypanocidal treatment: age at administration, drug, dose and duration.
- Follow-up: duration (months), diagnostic tests (number and type of laboratory tests used), quality control and comparison group.

Numerical data needed to conduct statistical analysis was abstracted into a worksheet by two reviewers (YS and KNR). Differences were resolved through discussion.

One of the review authors (YS) entered and organized data and citations in Review Manager Software [17]. We contacted the original authors of included studies to ask for any key information that was not included in their published report, whenever possible.

The protocol was registered at PROSPERO, an international prospective register of systematic review protocols (http://www.crd.york.ac.uk/PROSPERO, Registration Number CRD42012002162). The protocol for this systematic review and supporting PRISMA checklist are available as supporting information; see S1 Protocol and S1 Checklist.

### Risk of Bias Assessment

Two reviewers (YS and EH) evaluated the risk of bias of each included study independently using a pre-designed data extraction form with the principal domains of bias. The assessment of risk of bias for randomized and quasi-randomized trials was based on the Cochrane Collaboration’s tool [18]. The assessment of risk of bias for cohort studies was based on the guidelines developed by Hayden et al. [19].

We considered the following principal domains: selection, detection, attrition, confounding bias, and statistical analysis (excluding control for confounding). The risk of bias was rated as: low, high or unclear.

### Statistical Analysis

When collecting the numerical data, we realized that our outcomes of interest were presented as percentages, particularly reported at the end of the study follow-up. The total number of included subjects and the exact time of outcome assessment after treatment were not clearly stated in many of the included studies. In view of these limitations, we decided to conduct an exploratory analysis of data. We developed scatterplots of the percentage of participants with positive parasitological or negative serological outcomes (Y axis) versus posttreatment follow-up time in months (X axis). The dots represented the reported result from included studies. We applied the lowess curve to fitting a smooth curve to the points in the scatterplot [20]. The purpose of the curve was to summarize the central tendency of the Y variable’s distribution at different locations within the X variable’s distribution. The fitted curve was obtained empirically without requiring any specification of the relationship between the variables. Another advantage of the lowess procedure is that the smooth curve is more likely to track the more concentrated areas of data points, weighting down the outliers in the scatterplot.

We also defined eight intervals in months that are relevant for the clinical monitoring of patients after treatment: [0 to 3), [3 to 6), [6 to 12), [12 to 24), [24 to 144), [144 to 264), [264 to 384), and [384 to 504). Based on the available data, we estimated the weighted mean percentage for each outcome by using the inverse of variance, and the level of heterogeneity at each interval of time.

We measured heterogeneity by I^2^ as follows: low heterogeneity (I^2^ less than 25%), moderate heterogeneity (I^2^ between 25–75%) and high heterogeneity (I^2^ greater than 75%) [21].

### Additional Analyses

At the protocol stage, we planned to group data based on key factors that could affect treatment response in the chronic phase of *T*. *cruzi* infection. The subgroup categories included the age of subject at treatment (children versus adults), type of serological test (conventional serology versus non-conventional, in house under research serology reactions), duration of follow-up (less than five years versus equal or more than five years), and the region where the patient was born and assumed as the place where the infection was acquired.

We compiled the region data into three groups: 1) Argentina, Bolivia, Chile, Paraguay (Tc V), 2) Brazil (Tc II) and 3) other countries (Tc I). For studies involving different settings, we considered the country from which the majority of study participants came unless data could not be disaggregated.

We performed additional analysis according to the risk of bias assessment for each study as well.

## Results

### Included Studies

**Fig 1:**
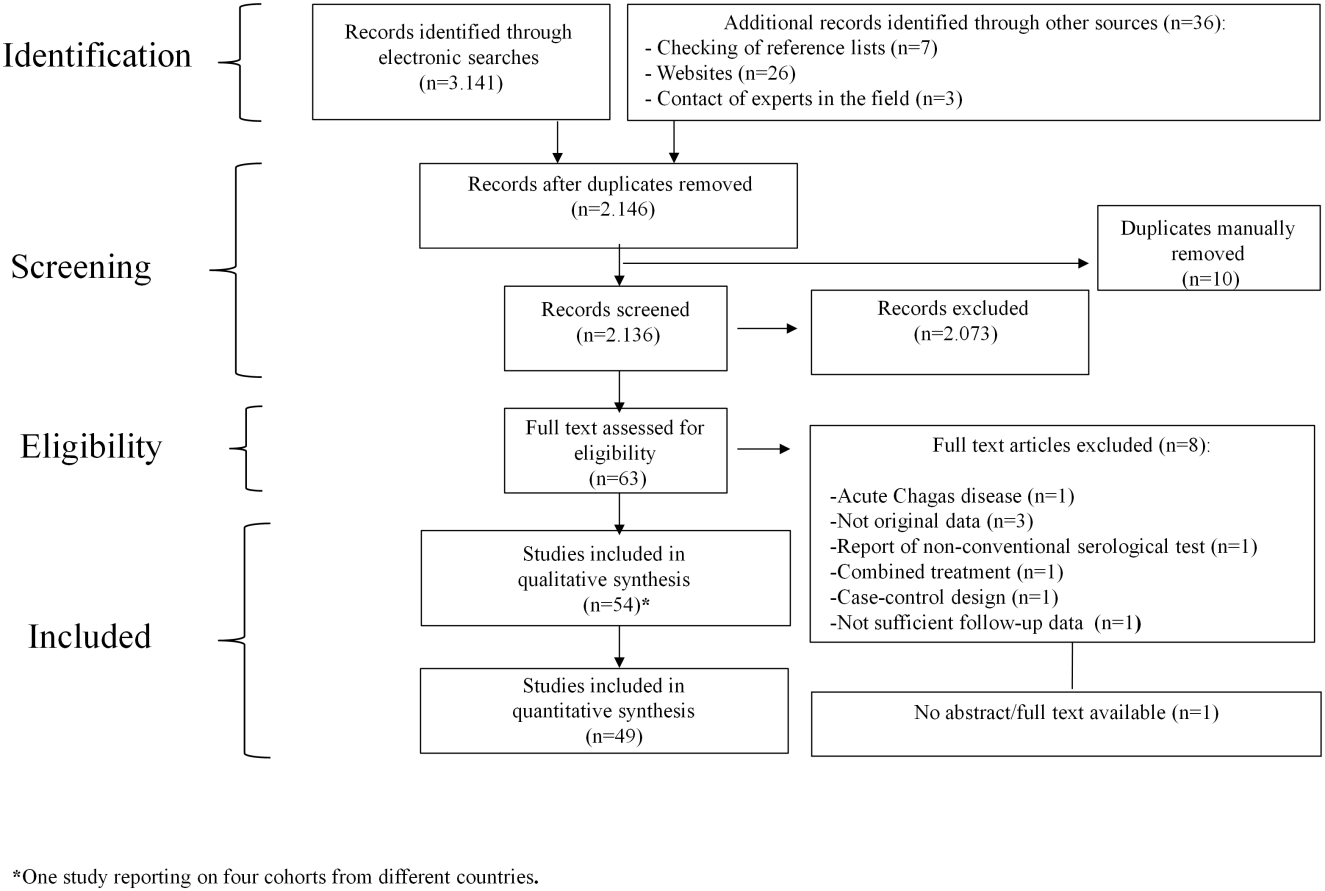
PRISMA flow diagram. The following diagram maps the number of records identified, included and excluded at different phases of the systematic review.

A total of 2,136 citations were screened (Fig 1). Fifty four out of 63 potentially eligible studies met our inclusion criteria. Six were RCTs [22–27], and 48 were observational studies [28–75]. The baseline characteristics of each included study are outlined in S1 Table.

The majority of the included studies were published after the decade of 1990s (83%). Among these, eight studies (18%) were published before 2000, twenty seven (60%) during the period 2000–2010 and ten (22%) after 2010. Most of the cohorts of subjects with chronic *T*. *cruzi* infection were born in countries within the South America region, mainly in Brazil (19 cohorts) and Argentina (14 cohorts).

Regarding the endemicity of study settings, twenty nine studies (54%) were conducted in endemic areas with risk of vectorial transmission of Chagas disease [23–27,30,33,34,40–43,45,48–51,53,54,56–59,62,64,65,68,71,73]. This information was not clearly stated or could not be inferred in five studies [38,46,67,74,75].

Twenty studies (37%) included adults [22,29,31,33,35,36,38,43,44,46,48,50,52,61,66, 69,70,73–75], sixteen studies (30%) included only children [23–25,27,32,41,54,49,51,54–56,59,62,64,65], and thirteen (24%) included both children and adults [28,30,34,37,39,40,42,47,57,60,63,68,72]. The age of study sample was not clearly reported in five studies (9%), but it can be assumed that they involved adults as well [26,53,58,67,71].

Thirty two studies (59%) used benznidazole [22–25,27,30,32–36,38–46,48–54,56,57,65,66,69] while nine studies (17%) used nifurtimox [29,31,59,64,71–75] and thirteen (24%) used both trypanocidal drugs [26,28,37,47,55,58,60–63,67,68,70].

The total duration of treatment was 30 days in five studies (9%) [26,36,44,52,66], between 30 and 59 in sixteen studies (30%) [28–30,35,37,38,40,41,45,47,55,57,60,61,67,69] and 60 days for twenty four studies (44%) [22–25,27,31–34,39,42,43,46,48–51,53,54,56,59,64,65,68]. Eight studies (15%) provided prolonged treatments [58,62,70–75]. One study did not report on treatment duration [63]. The dose of trypanocidal drug was not similar to current recommendations in five studies (9%) [31,64,70,74,75]. One study did not report the dose used [63].

Seven studies (13%) reported on parasitological outcomes [24,31,48,58,64,70,71], sixteen (30%) on serological outcomes [23,27,28,36,41,44–46,52,55–57,63,69,74,75], and thirty one (57%) provided results for both parasitological and serological outcomes during the follow-up [22,25,26,29,30–35,37–40,42,43,47,49–51,53,54,59–62,65–68,72,73].

The way of reporting the duration of follow-up after treatment varied among included studies (the minimum and/or the maximum, mean and median) and it ranged from one month to 24 years.

Eight studies were excluded [76–83], and details are provided in S2 Table. One study was not available [84]. We identified four potentially eligible ongoing studies [85–88].

### Synthesis of Results

Five out of 54 included studies were not included in the quantitative analysis [33,59,63,64,67]. The reasons were: a) no suitable numeric data were reported or could be inferred [33,63], and b) mixed cohort of subjects with acute and chronic *T*. *cruzi* infection [59,64,67].

#### Positive parasitological outcomes

High variability in positive parasitological and molecular tests was found before treatment because some included studies considered it an inclusion criterion.

**Fig 2:**
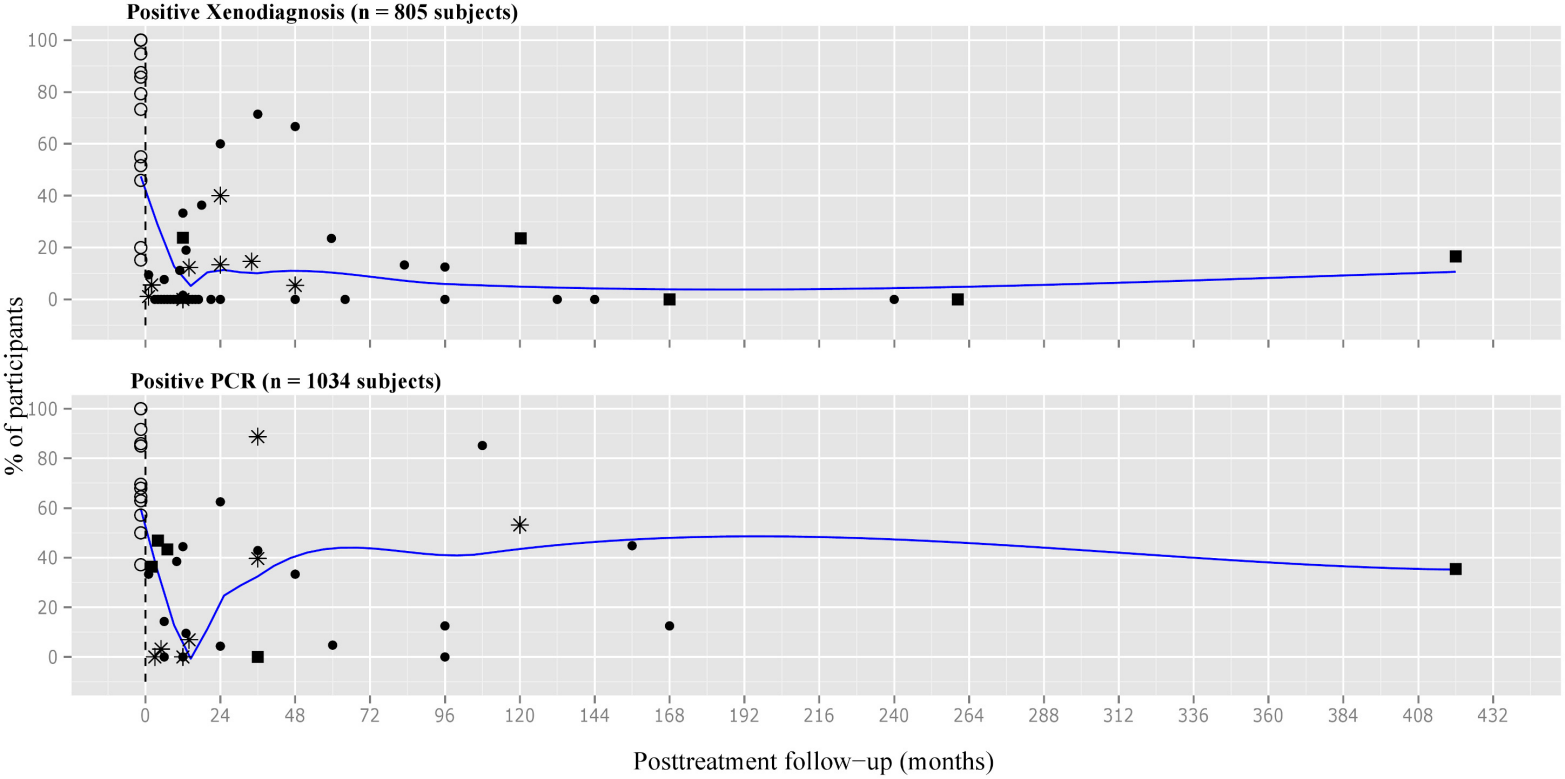
Scatter plot of percentages of positive xenodiagnosis and PCR in treated subjects with chronic *T*. *cruzi* infection. Number of participants: ● less than 30, ■ 30 to 50, * more than 50. PCR = polymerase chain reaction.

For xenodiagnosis, the average initial percentage for positivity was estimated at 50%. The administration of treatment resulted in a sharp curve descending towards 5% at 12 month, followed by a new increase of 10% at 24 months of follow-up. Afterwards, the curve went down to around 5% and returned to 10% many years later (Fig 2).

As for positive xenodiagnosis, a markedly decrease of the curve for hemoculture was observed during the first twelve months after treatment.

Regarding positive PCR, the average percentage before treatment was 60%. High variability of data during the first 48 months of follow-up was observed. After the early decrease of the curve for positive PCR, there was an increase of positivity to 40% at 48 months posttreatment and thereafter (Fig 2).

**Fig 3:**
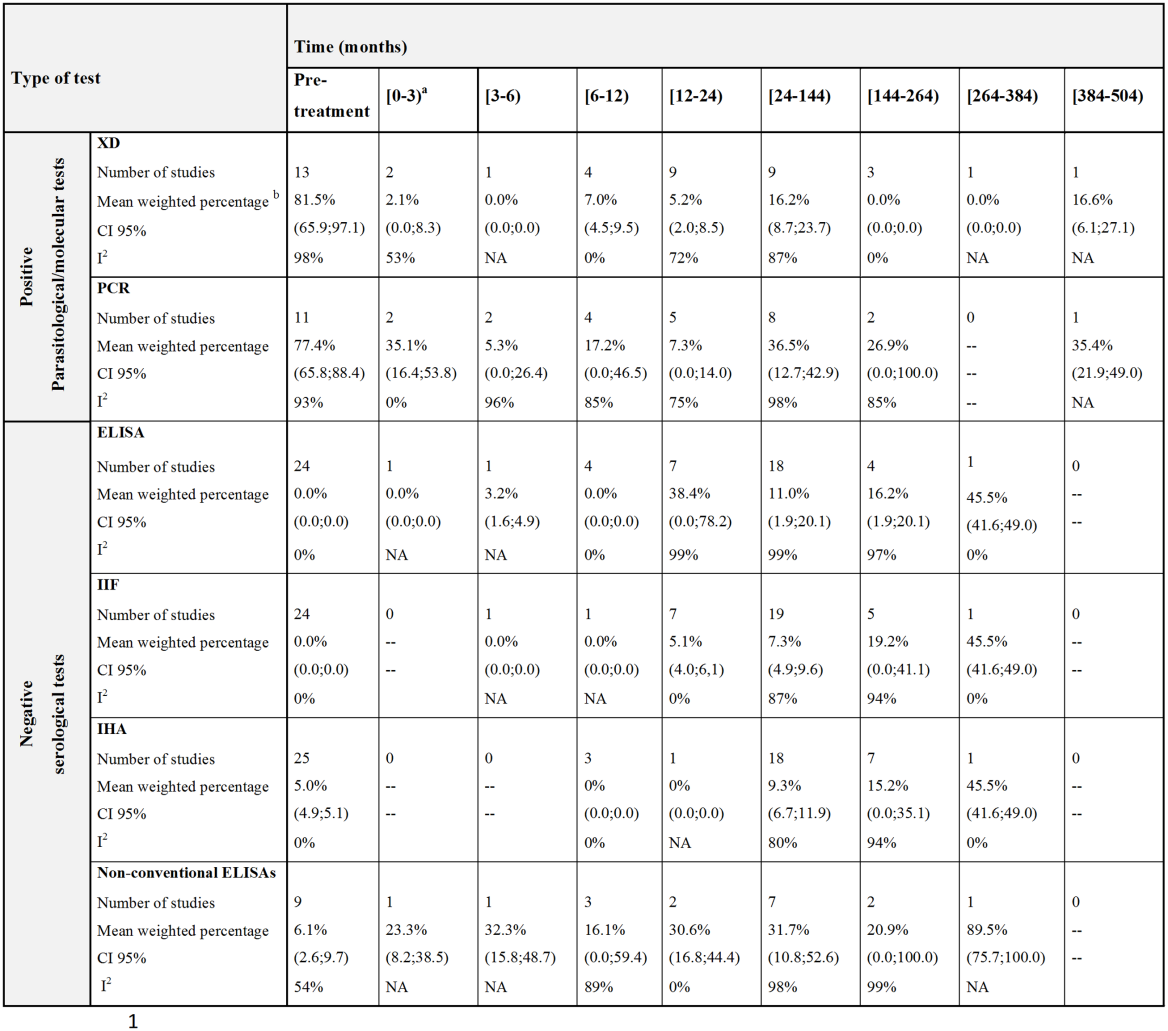
Mean weighted percentages for positive parasitological and negative serological tests in subjects with chronic *T*. *cruzi* infection before and after treatment.

The rates of treatment failure determined by positive xenodiagnosis and PCR at different intervals of the follow-up could be as high as 16% and 36%, respectively (Fig 3).

CI = confidence interval, ELISA = enzyme-linked immunosorbent assay, IIF = indirect immunofluorescence, IHA = indirect hemagglutination assay, NA = not applicable, PCR = polymerase chain reaction, XD = xenodiagnosis.

^a^ The use of a parenthesis indicates that the last number of the corresponding interval is not included. ^b^ The mean weighted percentage was estimated by the inverse of variance.

#### Negative serological outcomes

The scatterplot for negative ELISA test showed high variability of data, particularly between 12 and 36 months after treatment. The curve went up to 5% at 12 months, 10% at 24 months, and around 15% at 48 months. In the long-term follow-up, it almost reached 45%.

**Fig 4:**
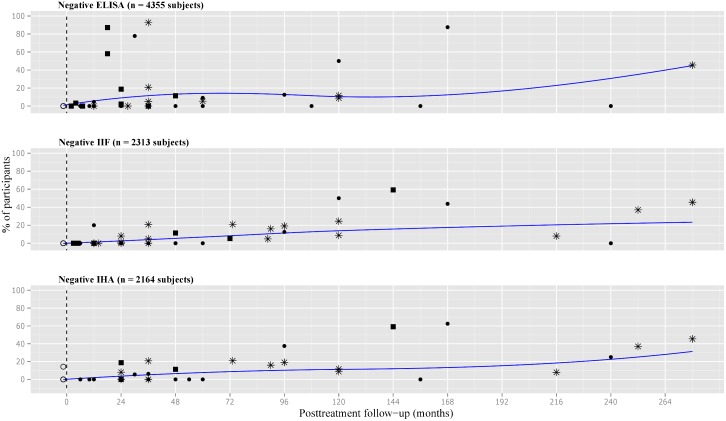
Scatter plot of percentages of negative conventional serological tests in treated subjects with chronic *T*. *cruzi* infection. Number of participants: ● less than 30, ■ 30 to 50, *more than 50. ELISA = enzyme-linked immunosorbent assay, IIF = indirect immunofluorescence, IHA = indirect hemagglutination assay.

The pattern of response to treatment based on IIF and IHA showed a similar trend. However, the depicted curve for negative IIF touched the 10% at 96 month of follow-up. In the very long-term, the curve for seronegativization reached 20% and 30% for IIF and IHA, respectively (Fig 4).

Details about negativization of serological tests at different time intervals after treatment are shown in Fig 3. Based on the data from chronically infected and treated subjects living in Santa Fe, Argentina [37], the best percentage of cure was 45%. Heterogeneity was always higher for ELISA in comparison to IIF and IHA tests.

The smoothed plotting for negative CF showed an ascendant curve towards 20% at 96 months of follow-up. Only four studies reported on negative DA-2ME, outlining a curve that reached 50% of negativization at 144 months posttreatment.

**Fig 5:**
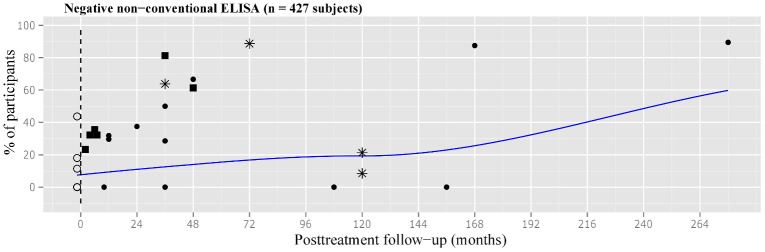
Scatter plot of percentages of negative non-conventional ELISA test in treated subjects with chronic *T*. *cruzi* infection. Number of participants: ● less than 30, ■ 30 to 50, *more than 50. ELISA = enzyme-linked immunosorbent assay.

The scatterplot for negative non-conventional ELISA tests was more informative during the first 48 months after treatment. The smoothed curve depicted a rising trend for negativization from around 8% up to 20% at 72 months after treatment. Negativization reached 60% in the very long-term follow-up (Fig 5).

The level of heterogeneity among the studies reporting on this outcome was also high (Fig 3).

**Fig 6:**
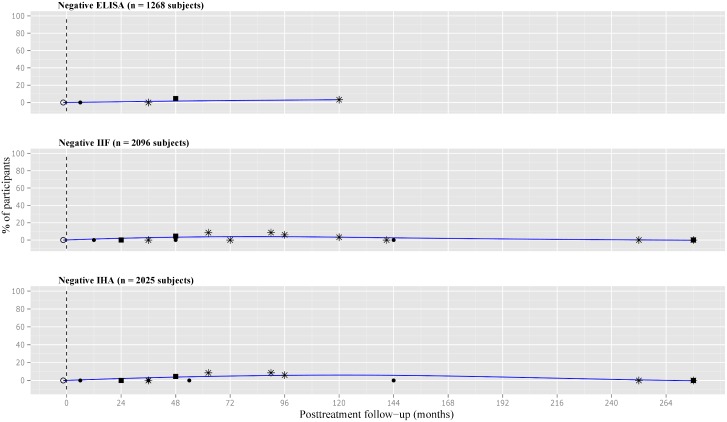
Scatter plot of percentages of negative conventional tests in subjects with chronic *T*. *cruzi* infection. Number of participants: ● less than 30, ■ 30 to 50, *more than 50. ELISA = enzyme-linked immunosorbent assay, IIF = indirect immunofluorescence, IHA = indirect hemagglutination assay.

Scatter plots for conventional serology in controls were developed. No seronegativization was reported in the eight studies [25,27,28,36,37,44,47,50] that measured anti-*T*. *cruzi* antibodies by ELISA in non-treated subjects. The curves in controls for negative IIF [25–28,36–38,44,47,52,55,60,61,66,69,72,73] and negative IHA [25,27,28,36,37,44,47,50,52,55,61,66,69,70,72,73] were initially flat but reached 2–3% at 72 months of follow-up (Fig 6).

Data for non-conventional ELISA in non-treated subjects showed highest rate of spontaneous negativization, i.e., 5% at 48 months and 10% at 72 months of follow-up.

For CF performed in untreated subjects [26,52,66,67,69,72], a tendency towards negativization of around 5% was found between 60–96 months of follow-up.

### Risk of Bias across Studies

**Fig 7:**
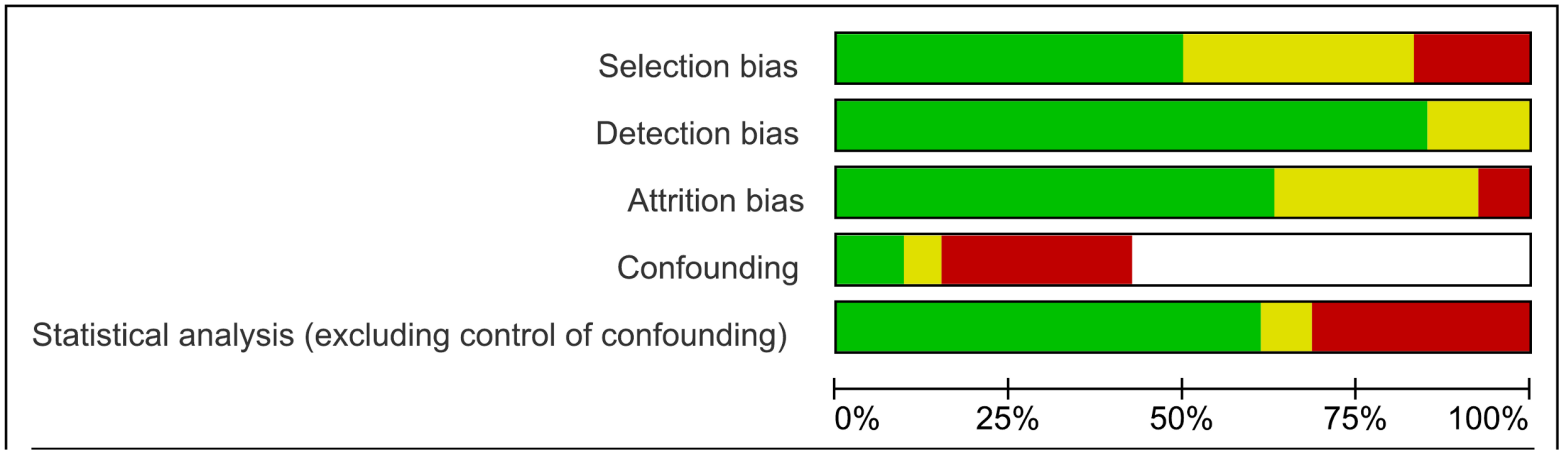
Risk of bias graph: review authors' judgments about risk of bias items presented as percentages across all included studies. Red: high risk, green: low risk, and yellow: unclear risk. The white strip for confounding corresponds to twenty seven follow-up studies without a control group, one RCT from Brazil [27] and two follow-up studies of this trial [23,24], one RCT from Argentina [25], and one RCT conducted in Spain [22] for which this risk of bias was rated as "not applicable".

Fig 7 summarizes the percentage of studies meeting each of the risk of bias domains.

See also S1 Fig with description of risk of bias assessment of each included studies.

- Cohort studies (n = 48)

The risk of selection bias was rated as low in twenty two (46%) studies, unclear in seventeen (35%) and high in nine (19%) studies.

The risk of detection bias was low in forty one (85%) studies and unclear in seven (15%) studies. Eight studies mentioned internal quality control measures for serological tests [30,31,34,41–43,45,54]. In two studies, 10% of positive and negative samples were sent to the reference laboratory for external quality control [41,45]. Positive and negative sera for Chagas disease were used as controls in five studies [30,34,39,42,63]. Different measures to avoid the risk of cross-contamination or PCR artifacts were reported in seven studies [22,33,40,50,53,59,64].

The risk of attrition bias was low in twenty seven (56%) studies, unclear in seventeen (36%) and high in four (8%).

Among the twenty cohort studies comparing a treated versus a non-treated group, the risk of bias for confounding was high in fifteen (75%) and low in five (25%).

The lack of explanations about the statistical methods used to perform the analysis of data was considered as high risk of bias in seventeen (35%) studies. It was rated as unclear in three studies.

- Randomized controlled trials (n=6)

Selection and detection bias was rated as low in five trials [22–25,27] and unclear in one [26]. The risk of attrition bias was rated as low in all included RCTs studies.

### Additional Analysis

We performed additional analysis based on the country of origin of study participants and risk of bias of included studies for all our outcomes of interest.

For xenodiagnosis and PCR, the scatter plots showed different patterns of response depending on the country where the studies were conducted (S2 Fig). Brazilian studies presented the highest rates of treatment failure, i.e., between 40% and 60%. Two studies were excluded from this analysis given that the required information was not reported [39,71].

Although the overall pattern of response observed when studies were grouped according to their risk of bias were parallel, there were some differences in the rate of positivity of xenodiagnosis and PCR tests after twelve months of treatment (S3 Fig). Studies judged as low risk of bias showed lower rates of treatment failure.

The scatter plots for ELISA, IIF and IHA tests showed similar trends for studies conducted in South America (S4 Fig). It might be inferred that the country of origin may not contribute to heterogeneity across studies, particularly before 120 months of follow-up. Beyond this period, the percentage of seronegativization identified was higher in studies from Argentina and Paraguay, i.e., around 40–50%, in comparison with studies conducted in Brazil. Two studies carried out in children from Guatemala and Honduras [41,45], showed the highest rate of seronegativization for ELISA test during the short-term follow-up. One study was not considered for this analysis because the country of origin could not be determined [39].

Studies judged as low risk of bias showed better rates of seronegativization, particularly for ELISA and IIF tests (S5 Fig). The curve for negative ELISA reached 40% at 48 months and then decreased to around 20% at 120 months after treatment. Afterwards, the scarcity of data precluded any plausible interpretation. In this regard, one study conducted in Argentina reported 85% of negativization in fourteen out of sixteen women who received benznidazole during infancy [43]. The curve for IIF test went up slowly after 48 months of treatment and reached 60%. No major differences were observed according to study quality for IHA test.

Regarding the age at treatment, we explored its potential impact for negative serological outcomes. In addition to the five studies that were not included in the statistical analysis, it was not possible to extract the data for different age groups in the following twelve [28,30,34,37,39,40,42,47,57,60,68,72]. The smoothed curves based on available data for conventional serology suggested that seronegativization occurs earlier in children compared to adults. This effect cannot be extrapolated beyond 120 months posttreatment (S6 Fig).

## Discussion

Since the 1990s, the lack of adequate parameters to determine the effectiveness of trypanocidal drugs in subjects with chronic *T*. *cruzi* infection has encouraged researches to conduct systematic reviews of treatment effects on clinical, parasitological and serological endpoints. In this framework, the WHO recommends to offer trypanocidal drugs to all subjects with chronic *T*. *cruzi* infection. The success of the treatment is determined by the disappearance of anti-*T*. *cruzi* antibodies, while therapeutic failure is determined by parasite detection in blood.

The long-term follow-up hampers the practical usefulness of conventional serology to assess treatment success in the chronic phase of Chagas disease [89]. This issue could be partly responsible for the reluctance of health workers to prescribe specific treatment to chronically infected patients.

The better understanding of the kinetics of parasitemia and of specific antibodies after treatment may help to develop algorithms of clinical management for chronic *T*. *cruzi* infection. It may also enrich the discussion about the conceptual meaning of favorable response, surrogates of treatment response and need for new marker tools [89] to envisage treatment success in the short-term follow-up.

The kinetics of seronegativization is variable due to changes of reactivity against specific antibodies among serological assays. This fact was observed in two RCTs included in this review [25,27].

Non-conventional ELISA tests are usually performed with a recombinant antigen making them less sensitive. It has been recognized that some are not useful for diagnosis but worthy for monitoring therapeutic response.

The presence of *T*. *cruzi* is now accepted as a trigger for the pathophysiological phenomenon of the chronic infection, as well as a key factor for its magnitude and persistence [90]. From this standpoint, a trypanocidal effect is relevant for the cure of subjects [91], the potential impact on decreased morbidity and mortality [52,87] and the prevention of vertical transmission of Chagas disease [43,92].

The level of susceptibility of different lineages and how this factor may interfere with treatment response are unknown, although some results suggest there is an impact [93].

### Summary of Main Results

The smoothed curves for positive xenodiagnosis and PCR were characterized by a sharp decrease at twelve months after treatment. Afterwards, the curve for positive xenodiagnosis has a point of inflection showing a progressive increase towards 10% at 24 months, and then it went down again towards 5%. For PCR, the curve for positivity as average reached 40% at 48 months of treatment and it did not decrease after that.

The treatment failure’s rate found in our analysis should be considered a qualitative result. It was a bit higher than the rate for real-time PCR assay reported in a recent trial carried out in Spain [22] and the preliminary analysis of a recent trial using benznidazole [94]. Concerning this issue, it has recently been established that the parasite load associated with a positive PCR remains low for long periods of time (personal communication of the principal author), calling into question the real value of parasite load reduction as a marker for treatment response [89].

The curve for negative ELISA went up 15% at 48 months and 45% in the very long-term follow-up. The patterns of response to treatment based on IIF and IHA were similar but reached 20% and 30%, respectively. The observed differences may relate to singular attributes of serological techniques for catalyzing the antigen-antibody reaction.

The evolution of serological results in subjects with chronic *T*. *cruzi* infection who did not receive trypanocidal treatment was not favorable. The spontaneous seronegativization rate of around 3% could be related with an efficient immune response getting a “spontaneous cure”.

Heterogeneity was marginally assessed by additional analysis. The country of origin of study subjects might impact on parasitological and molecular endpoints, while the overall risk of bias of included studies might contribute to variability of serological results. We pointed out that most of the Brazilian studies reporting on xenodiagnosis were judged as having a high risk of bias.

The age at treatment is a variable to consider when assessing the success of trypanocidal drugs.

### Limitations

Three pilot tests of search strategies were carried out in MEDLINE to explore the potential sensitivity and specificity of the electronic searches by one review author (YS). Taking into account these results and following the advice of the trial search coordinator of the review team, we decided not to use a filter for prognosis studies. We assume that the risk of publication bias is low but cannot be ruled out.

We were not able to discard the following exclusion criteria with absolute confidence: exposure to any vectors in the past six months and blood transfusions in the past twelve months.

In defining the research question for this review, we admitted that differences in the way the studies were conducted could impact on our findings. Each included study had its own eligibility criteria and was conducted in different contexts. Populations differed in the age at treatment, the performance of laboratory test (particularly for PCR, a molecular technique that was first use for Chagas disease in 1992), how cut-off points were defined, and time points for outcome measurements. This clinical heterogeneity can be the cause of the high statistical heterogeneity. In addition, unmeasured differences due to confounding could have also contributed. Potential confounding factors are the geographical region where the circulating *T*. *cruzi* lineage is diverse [95], the age of participants at treatment, and return to endemic area.

Poor reporting was a major problem for quality assessment of included studies. To address this limitation, we tried to contact the primary investigators asking for their help in providing missing data and clarifications, particularly related to selection bias.

The way of reporting aggregated data did not allow us to pursue the planned statistical strategy. For instance, we explored the effect of the duration of trypanocidal treatment. The categories for the subgroups feasible to create based on reported data were < 60 days versus ≥ 60 or more days of treatment. Ten studies were not included in this analysis because they used more than one trypanocidal treatment schedule [26,28,37,47,55,58,60,62,68,70]. We were cautious when presenting additional analysis based on aggregated data.

## Conclusions

We acknowledge that there is dynamic pattern of response based on parasitological, molecular serological tests in subjects chronically infected with *T*. *cruzi* after treatment. Our findings suggest a trypanocidal effect in the long-term follow-up.

Further research is needed to explore potential sources of heterogeneity and to conduct reliable subgroup analysis.

### Implications for Future Research

When faced with a research question vis-à-vis the potential association between time and event, investigators have to choose an appropriate strategy to resolve the matter.

To answer our research question, we consider the following factors: the natural history and pathophysiology of the disease, the time elapsing between treatment administration and the event of interest, and the available resources. In this scenario, the next step will be to conduct a more specific type of meta-analysis: an Individual Participant Data Meta-Analysis (IPD MA). Rather than extracting aggregated data from publications, the original study data will be sought directly from the principal investigators. These data could then be re-analyzed centrally and combined, if appropriate.

The positive thesis is that many of the ostensible benefits of longitudinal research may be obtained by carefully designed IPD meta-analysis, at substantially reduced time and cost. The possibility of performing additional analysis based on IPD may overcome pragmatic constraints such as the long time elapsed between exposure to trypanocidal treatment and the outcome of interest, the estimation of appropriate sample sizes for children and adults, and the need of adequate rates of follow-up.

For the purpose of our research, this approach may allow to have larger availability of data (increased sample size, additional follow-up data), better presentation of data (access to raw datasets, standardization of analyses across studies, checking of inconsistencies, etc.), and more informative and reliable subgroup analyses [96]. Among the advantages of this type of meta-analysis is the feasibility to perform additional analysis such as “time to event”. Understanding survival curves will help clarifying current thinking about treatment choices as well as about prognosis of treated subjects with chronic *T*. *cruzi* infection.

It is expected that new empirical evidence raised from IPD meta-analysis of follow-up studies would help to test our hypothesis and contribute to current knowledge about Chagas disease.

We updated the electronic searches on 26^th^ July 2015 to assess how many potentially eligible studies have been published since 2013. Among the 395 cites retrieved, we identified four new eligible studies [97–100]. Additionally, two studies have been recently identified by contacting their principal investigators [101, 102]. This new evidence is consistent with our findings and will be included in future updates of the systematic review and meta-analysis.

## Supporting Information

S1 Checklist PRISMA checklist. Checklist for reporting of systematic reviews and meta-analysis.
(DOC)

S1 Fig Review authors' judgments about each risk of bias item for each included study. Red: high risk, green: low risk, and yellow: unclear risk. The blank boxes for confounding corresponds to twenty seven follow-up studies without a control group, one RCT from Brazil [27] and two follow-up studies of this trial [23,24], one RCT from Argentina [25], and one RCT conducted in Spain [22] for which this risk of bias was rated as "not applicable".
(TIF)

S2 Fig Scatter plot of percentages of positive xenodiagnosis and PCR tests in treated subjects with chronic *T*. *cruzi* infection based on the country of origin. Red: Argentina, Bolivia, Chile and Paraguay, blue: Brazil, green: other countries. PCR = polymerase chain reaction.
(TIF)

S3 Fig Scatter plot of percentages of positive xenodiagnosis and PCR tests in treated subjects with chronic *T*. *cruzi* infection based on risk of bias. Red: low risk of bias, blue: high risk of bias. PCR = polymerase chain reaction.
(TIF)

S4 Fig Scatter plot of percentages of negative conventional serological test in treated subjects with chronic *T*. *cruzi* infection based on the country of origin. Red: Argentina, Bolivia, Chile and Paraguay, blue: Brazil, green: other countries. ELISA = enzyme-linked immunosorbent assay, IIF = indirect immunofluorescence, IHA = indirect hemagglutination assay.
(TIF)

S5 Fig Scatter plot of percentages of negative conventional serological test in treated subjects with chronic *T*. *cruzi* infection based on the risk of bias. Red: low risk of bias, blue: high risk of bias. ELISA = enzyme-linked immunosorbent assay, IIF = indirect immunofluorescence, IHA = indirect hemagglutination assay.
(TIF)

S6 Fig Scatter plot of percentages of negative conventional serological test in treated subjects with chronic *T*. *cruzi* infection based on age at treatment. Red: children, blue: adults. ELISA = enzyme-linked immunosorbent assay, IIF = indirect immunofluorescence, IHA = indirect hemagglutination assay.
(TIF)

S1 Protocol Pre-registered review protocol. (PDF)

S1 Table Characteristic of included studies (n = 54). Ag = antigen, AT-ELISA = chemiluminescent enzyme-linked immunosorbent assay with a trypomastigote mucin antigen, BZN = benznidazole, CF = complement fixation, CoML = complement mediated lysis, d = day, DA-2ME = direct agglutination with 2-mercaptoethanol, ELISA = enzyme-linked immunosorbent assay, ELISA-F29 = ELISA test using the F29 protein of *T*. *cruzi*, FC-ALTA-IgG = anti-live-trypomastigote antibodies, HA = hemagglutination assay, HC = hemoculture, IIF = indirect immunofluorescence assay, IF = immunofluorescence, IHA = indirect hemagglutination assay, IRA-ELISA = ELISA with an individual recombinant antigens, kg = kilogram, mg = milligram, mo = months, multiplex serological assay = a panel of 14 recombinant *T*. *cruzi* proteins in a Luminex-based format, NFTX = nifurtimox, PCR = polymerase chain reaction, rCRP = recombinant complement regulatory protein, RCT = randomised controlled trial, rt-PCR = real time PCR, RAM-ELISA = ELISA with a recombinant antigen mixture, SAPA = shed acute-phase antigen, TESA-blot = immunoblot assay, XD = xenodiagnosis, y = years. ^1^conventional “in house” serological tests (unless indicated otherwise). ^2^commercial conventional test. ^3^ non-conventional test.
(PDF)

S2 Table Table of excluded studies (n = 8). BNZ = benznidazole, d = day, kg = kilogram, mg = milligram, NFTX = nifurtimox.
(PDF)
