# Supplementary figures and images for: Course of Chronic Trypanosoma cruzi Infection after Treatment Based on Parasitological and Serological Tests: A Systematic Review of Follow-Up Studies
Source: PLoS One. 2015 Oct 5;10(10):e0139363. doi: 10.1371/journal.pone.0139363 (PMC4593559; doi:10.1371/journal.pone.0139363)

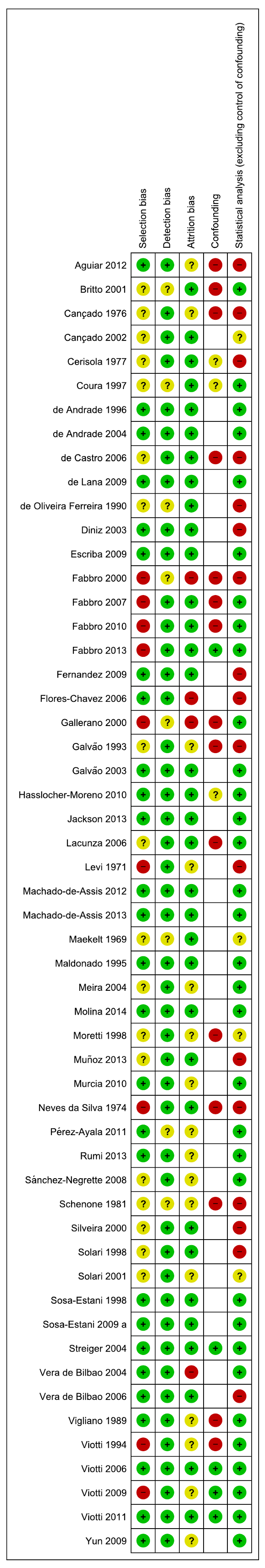

Supplement: S1 Fig — Red: high risk, green: low risk, and yellow: unclear risk. The blank boxes for confounding corresponds to twenty seven follow-up studies without a control group, one RCT from Brazil [27] and two follow-up studies of this trial [23,24], one RCT from Argentina [25], and one RCT conducted in Spain [22] for which this risk of bias was rated as "not applicable". (TIF) [file pone.0139363.s002.tif]

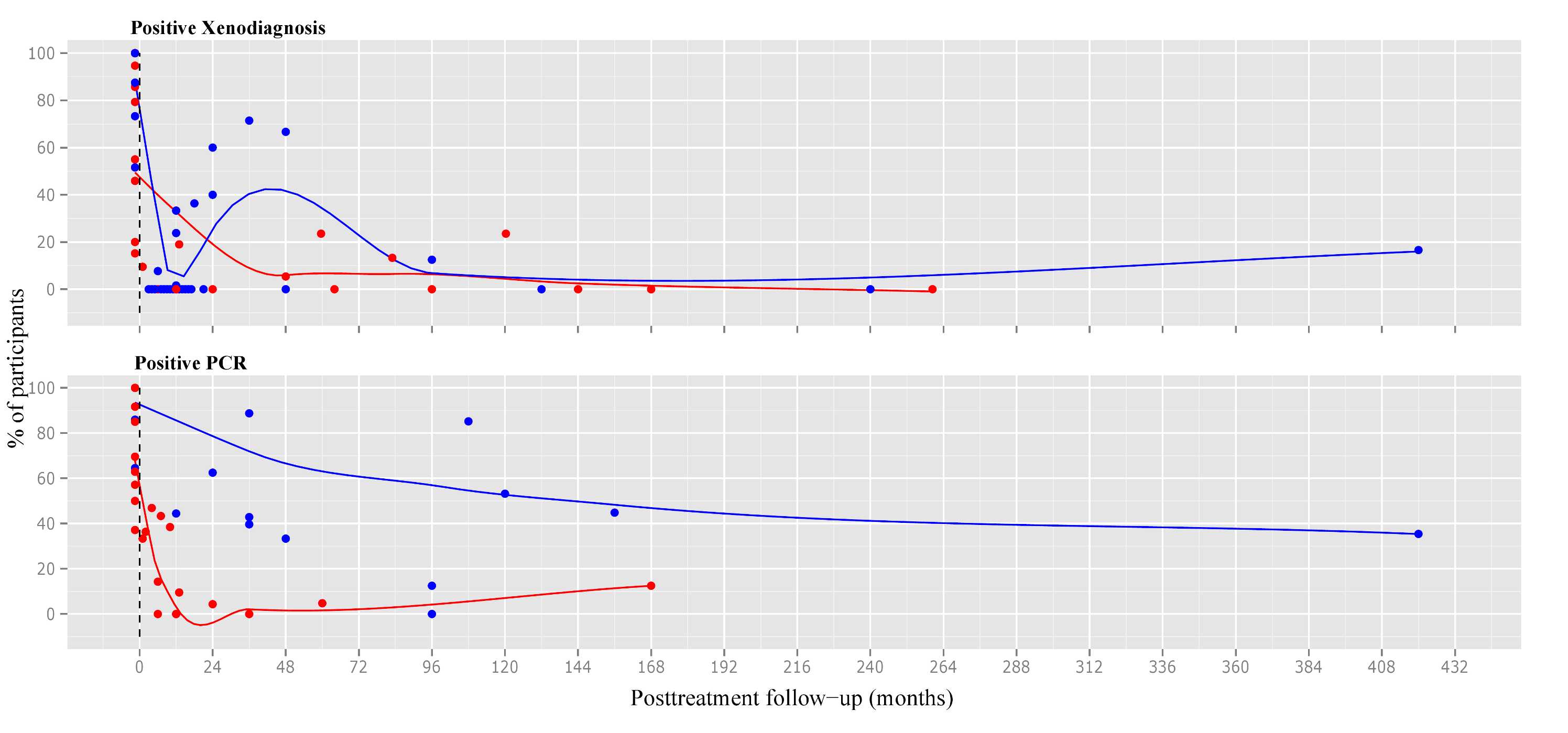

Supplement: S2 Fig — Red: Argentina, Bolivia, Chile and Paraguay, blue: Brazil, green: other countries. PCR = polymerase chain reaction. (TIF) [file pone.0139363.s003.tif]

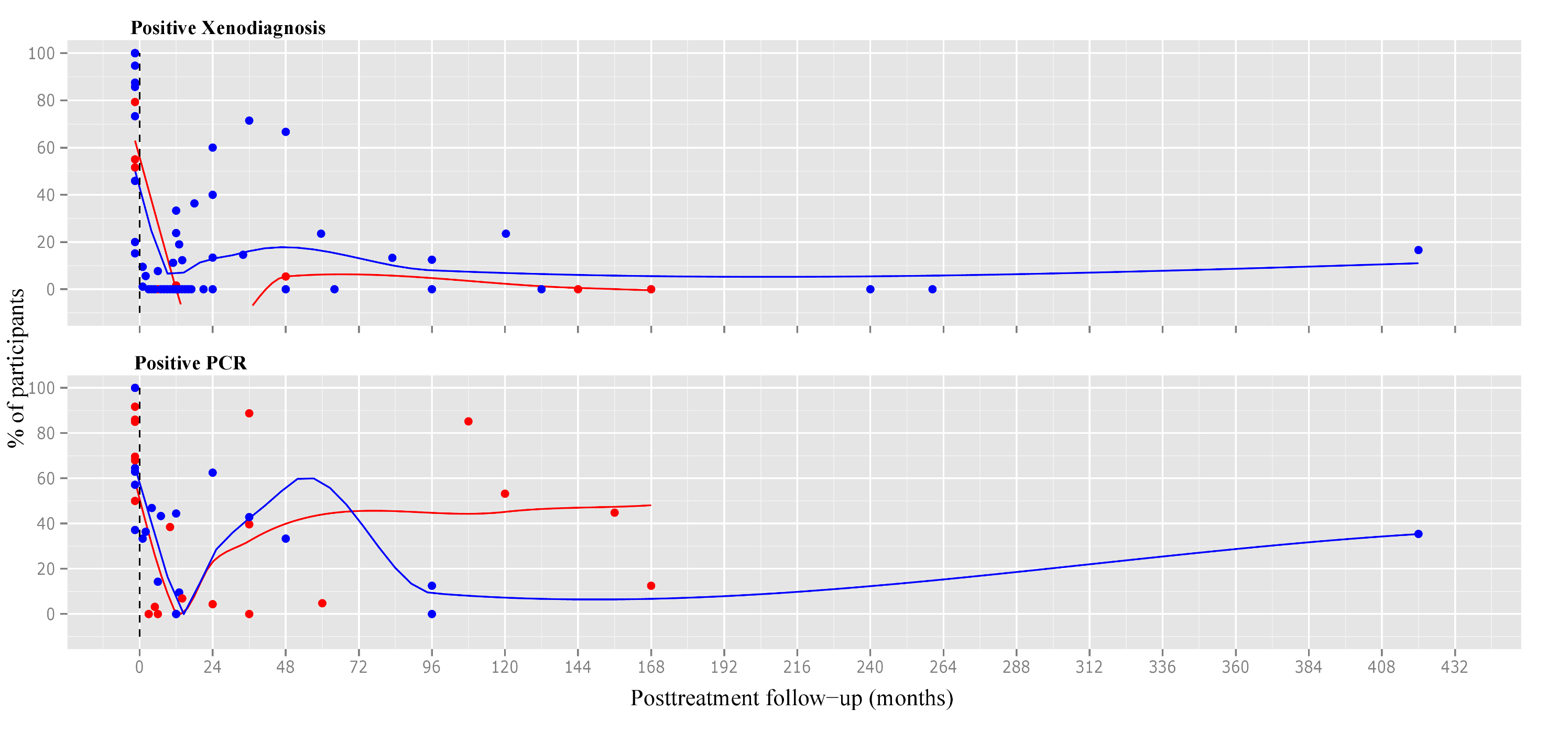

Supplement: S3 Fig — Red: low risk of bias, blue: high risk of bias. PCR = polymerase chain reaction. (TIF) [file pone.0139363.s004.tif]

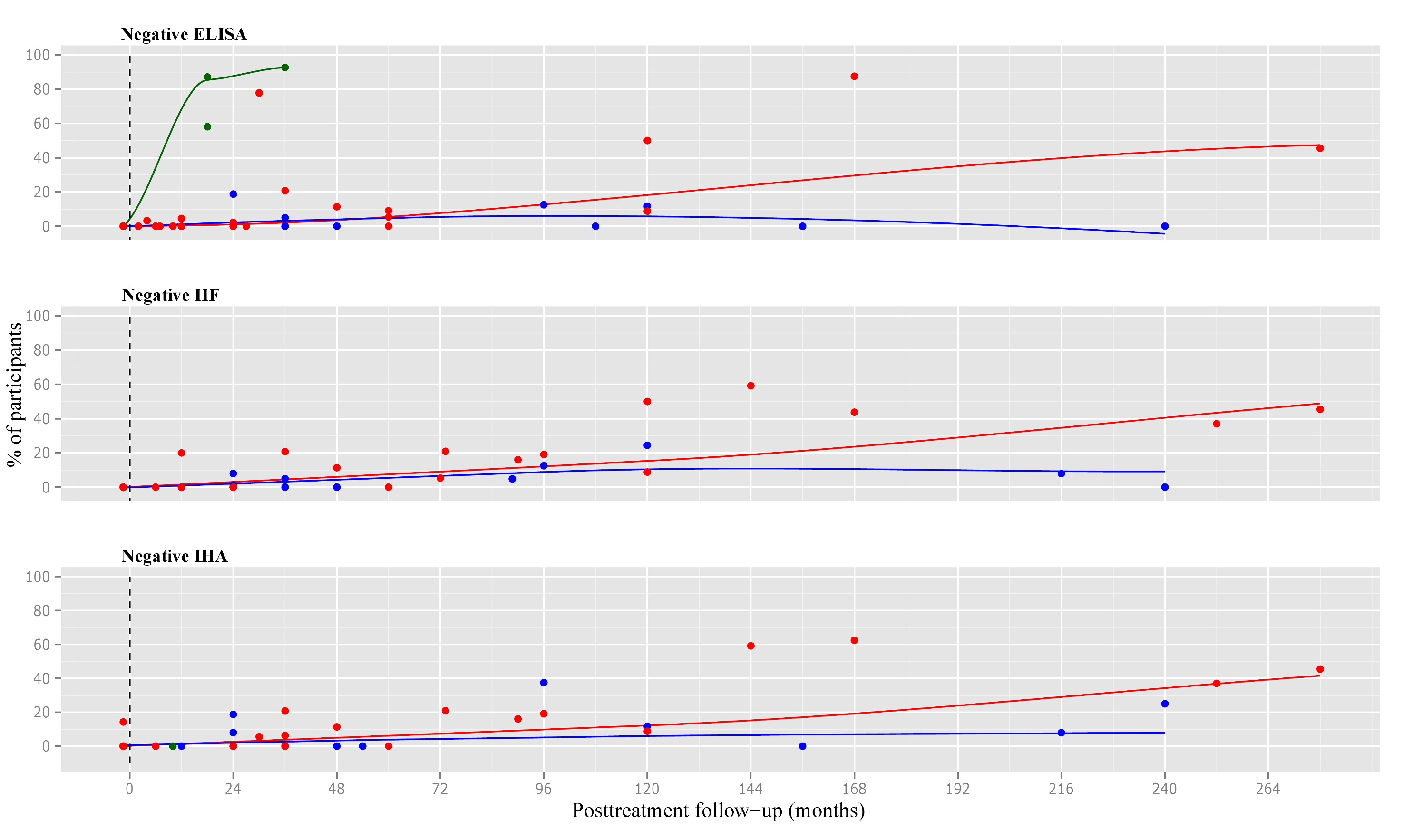

Supplement: S4 Fig — Red: Argentina, Bolivia, Chile and Paraguay, blue: Brazil, green: other countries. ELISA = enzyme-linked immunosorbent assay, IIF = indirect immunofluorescence, IHA = indirect hemagglutination assay. (TIF) [file pone.0139363.s005.tif]

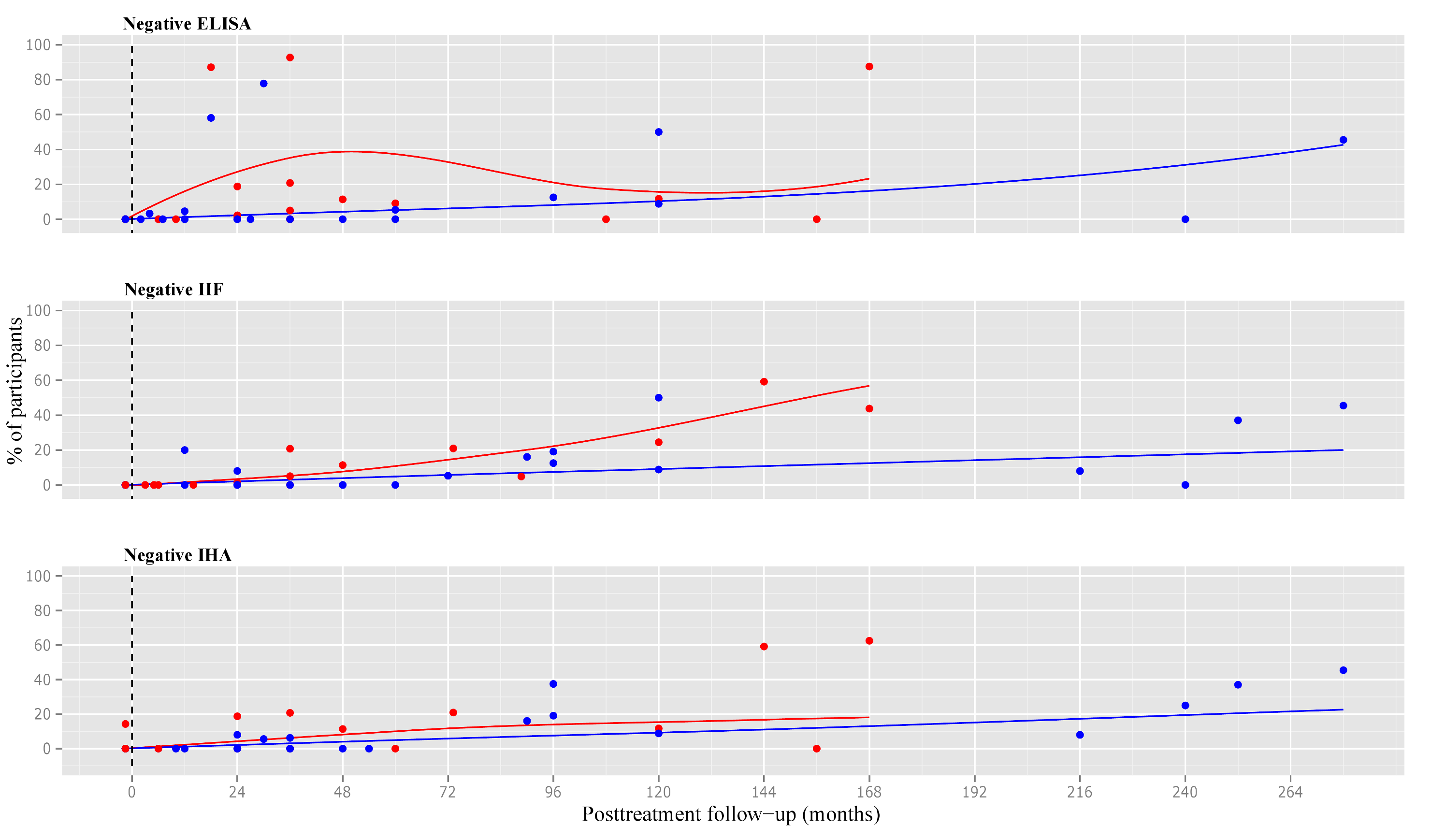

Supplement: S5 Fig — Red: low risk of bias, blue: high risk of bias. ELISA = enzyme-linked immunosorbent assay, IIF = indirect immunofluorescence, IHA = indirect hemagglutination assay. (TIF) [file pone.0139363.s006.tif]

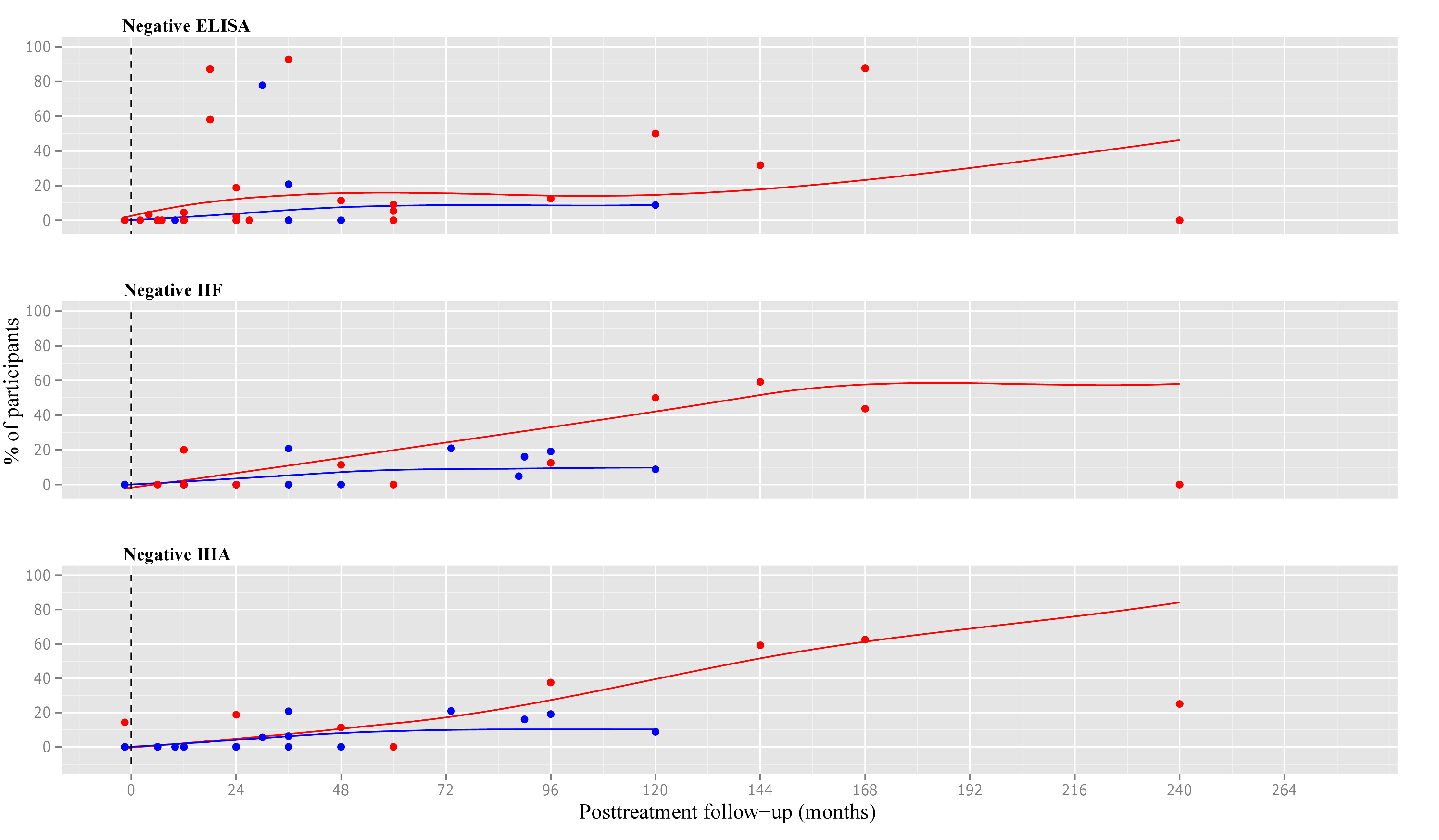

Supplement: S6 Fig — Red: children, blue: adults. ELISA = enzyme-linked immunosorbent assay, IIF = indirect immunofluorescence, IHA = indirect hemagglutination assay. (TIF) [file pone.0139363.s007.tif]
