# Supplementary material for: Course of Chronic Trypanosoma cruzi Infection after Treatment Based on Parasitological and Serological Tests: A Systematic Review of Follow-Up Studies
Source: PLoS One. 2015 Oct 5;10(10):e0139363. doi: 10.1371/journal.pone.0139363 (PMC4593559; doi:10.1371/journal.pone.0139363)
Supplement: S1 Protocol — (PDF) [file pone.0139363.s008.pdf]

## PROSPERO International prospective register of systematic reviews

# Course of chronic Trypanosoma cruzi infection after treatment based on parasitological and serological tests: a systematic review of follow-up studies

*Yanina Sguassero, Christine Christensen, Cristina Cuesta, Karen Roberts, Daniel Comandé, Agustín Ciapponi, Daniel Giordano, Elizabeth Hicks, Sergio Sosa-Estani*

## Citation

Yanina Sguassero, Christine Christensen, Cristina Cuesta, Karen Roberts, Daniel Comandé, Agustín Ciapponi, Daniel Giordano, Elizabeth Hicks, Sergio Sosa-Estani. Course of chronic Trypanosoma cruzi infection after treatment based on parasitological and serological tests: a systematic review of follow-up studies. PROSPERO 2012:CRD42012002162 Available from
http://www.crd.york.ac.uk/PROSPERO_REBRANDING/display_record.asp?ID=CRD42012002162

## Review question(s)

Our research aims to study "when" and "to what extent" the administration of trypanocidal drugs causes the negativization of serology tests commonly used to control subjects with chronic T. cruzi infection.

Our primary objective is to summarize what patterns of commonly used tests may predict response to treatment (success or failure) in the follow-up of subjects with chronic T. cruzi infection.

There are two secondary objectives. The first is to assess the disease course of treated subjects with chronic T. cruzi infection compared to subjects who were not treated. The last objective is to appraise the quality of available studies on parasitological and serological test response in chronic T. cruzi infection after treatment.

## Searches

-Electronic searches

The following databases will be searched: the Cochrane Database of Systematic Reviews (CDSR), Cochrane Central Register of Controlled Trials (CENTRAL), Database of Abstracts of Reviews of Effects (DARE), MEDLINE, EMBASE, LILACS, Clinicaltrials.gov and the WHO International Clinical Trials Registry Platform (ICTRP).

Where necessary, the search terms will be modified to suit the requirements of particular databases. No language limitations or publication date restrictions will be applied.

For studies with multiple publications, we will decide how to use the data on a case by case basis through discussion with the principal investigators.

The search strategy will include the following terms:

(Chagas Disease[Mesh] OR Chagas[tiab] OR Trypanosom*[tiab] OR Cruzi[tiab] OR T.Cruzi[tiab]) AND (Benzonidazole[Supplementary Concept] OR benznidazol*[tiab] OR Radanil[tiab] OR Nifurtimox[Mesh] OR nifurtimox[tiab] OR Lampit[tiab])

-Searching other resources

Reference lists

The reference lists of retrieved articles and relevant reviews will be checked for potentially eligible studies.

Correspondence

We will contact the author of potentially eligible studies to identify additional ongoing or unpublished research. We

will also contact the original authors of included studies to ask for any information that is not contained in their published report.

Key people from organizations focusing on infectious diseases will be contacted by email. These e-mails will introduce the plan for our review, and ask for help in identifying potentially relevant studies that we may have missed. The organisations we will intend to approach are listed below:

-The World Health Organization (www.who.int/tdr)

-The Pan-American Health Organization (www.paho.org/chagas)

-The UK Department for International Development (http://r4d.dfid.gov.uk/SiteSearch.aspx?q=chagas)

-Mundo sano fundation (www.mundosano.org)

Trials Registers

ClinicalTrials.gov (http://www.clinicaltrials.gov/)

MetaRegister of Controlled Trials (http://www.controlled-trials.com/mrct/)

WHO International Clinical Trials Registry (ICTRP) (http://apps.who.int/trialsearch/)

### Types of study to be included
Observational (cohort studies) and experimental (RCT, quasi-RCTs) designs will be included.

### Condition or domain being studied
Chagas disease is caused by the flagellate protazoa Trypanosoma cruzi (T. cruzi) and is endemic to 21 countries in the Americas, with more cases appearing globally as infected individuals migrate. In this context, Chagas disease threatens to expand exponentially, from rural to urban areas and endemic to non-endemic regions.

It has been estimated that 7 to 8 million people are chronically infected with T. cruzi and that 12,000 people die each year as a result. Moreover, 100 million of people are at risk of having the disease.

The disease is principally transmitted through vectors, namely triatomine bugs. These insects live in cracks and crevices of poor-quality houses, typically in rural areas. The parasite may also be transmitted through blood transfusions, organ donation, laboratory accidents, congenital transmission from an infected mother to child, or orally through food contaminated with insect or feces with parasites.

The disease evolves in two phases, acute and chronic, each of them with different clinical characteristics and diagnostic criteria.

The Acute Phase starts immediately after infection by any route of transmission and lasts between 1-3 months. Most people are asymptomatic in the Acute Phase, with approximately 8% of cases developing nonspecific symptoms such as prolonged febrile syndrome, adenomegalies, and diarrhea.

Subjects who do not receive etiological treatment during the Acute Phase go on to develop chronic infection, which begins when the presence of the parasite in the blood becomes undetectable through direct parasitological methods.

An absence of clinical symptoms or signs of visceral lesions characterize it. The indeterminate form (also called “Phase without demonstrated pathology”) may persist for the lifetime of the patient or it may progress into the Chronic Phase to a cardiac, digestive, neurological, or mixed form after 15 or 20 years. Long-term complications of chronic T. cruzi infection include heart failure, dysrythmias, and megacolon.

### Participants/ population
Adults and children with T. cruzi infection in the chronic phase who received adequate treatment and were followed

over time.

Chronic Chagas infection will be defined as T. cruzi infection confirmed with at least two positive serological tests, no exposure to any vectors in the past six months, and no blood transfusions in the past twelve months. If the case is congenital, the child is considered to be in the chronic phase after he/she has reached twelve months of age. Benznidazole 5-7mg/kg per day or nifurtimox 10-12mg/kg (infants) or 8-10mg/kg (adults) for 30-60 days will be considered adequate treatment.

### Intervention(s), exposure(s)

Parasitological or direct test:

- Hemoculture: Heparinized blood samples are incubated on media for a period of time to detect the presence of T. cruzi by culture.

-Xenodiagnosis: T. cruzi naïve Trypanosomas insects are exposed to the patient and after a period of time, the intestinal content of the insects is analyzed for the presence of T. cruzi.

-PCR: Detection of T. cruzi DNA using polymerase chain reaction (PCR).

Other parasitological tests such as the fresh drop, Strout method (visualization of T. cruzi organisms by light microscope in the sediment of the supernatant of non-heparinized blood after centrifugation) and micromethod (microscopic visualization of T. cruzi organisms in the buffy coat of blood samples after centrifugation), will not be considered because these tests are systematically negative during the chronic phase.

Serological or indirect test:

-IIF (indirect immunoflorescence)

-ELISA (enzyme linked immunosorbent assay)

-DA-2ME (direct hemagglutination with and without 2-mercaptoethanol)

-IHA (indirect hemagglutination assay), etc.

There are also non-conventional tests based on recombinant/synthetic and biochemically purified antigens, which have not been standardized or validated.

### Comparator(s)/ control

We will compare “chronic infected patients who received treatment” versus “chronic infected patients who did not receive treatment” (Secondary objective).

### Context

The following exclusion criteria for studies will be applied:

-Participants in the acute phase when they received treatment.

-Children aged 12 months or younger born to infected mothers.

-Immunocompromised participants.

-Pregnant women.

We will also consider the following definitions:

-Definition of chronic T. cruzi infection: serologically confirmed presence of T. cruzi (at least two positive serological tests) with an absence of exposure to any vectors in the past six months, and no transfusions received in the past 12 months. If the case is congenital, the child is considered to be in the chronic phase after he/she has reached

12 months of age.

-Definition of adequate treatment of chronic T. cruzi infection: treatment with adequate doses of trypanocidal drugs for 30-60 days.

-Nifurtimox: the recommend dose for children is 10 mg/kg of body weight per day and for adults is 8 mg/kg of body weight per day (maximum of 700 mg per day).

-Benznidazol: 5- 7 mg/kg of body weight per day.

-Definition of chronic T. cruzi infection cure: negative seroconversion (absence of antibodies specific to T. cruzi in the blood) or a continued decrease in titers of antibodies specific to T. cruzi. Based on previous research, a decreasing titer of antibodies should be at least 2 dilutions for IFI and 50% for ELISA.

-Definition of treatment failure in chronic T. cruzi infection (not cure): positive parasitological test indicating the persistence of the parasite.

-Definition of adequate follow-up period: minimum number of years of follow-up is equal or greater than the age of the patient when specific treatment was received (e.g. if a patient with chronic T. cruzi infection received treatment at 10 years of age, the minimum of follow-up should be 10 years). Nevertheless, the minimum duration of follow up is arbitrary based on previous research conducted in Argentina.

## Outcome(s)

### Primary outcomes

We will assess the following outcomes at different time points throughout the follow-up:

1.- Negative results of parasitological tests such as xenodiagnosis or PCR (clearance of parasitemia).

2.- Positive results of parasitological tests such as xenodiagnosis or PCR (persistence of parasitemia and treatment failure).

3.- Seroconversion, defined as a qualitative change to the serological reaction against T. cruzi from being “seropositive” to being “seronegative”, i.e., disappearance of T. cruzi antibodies (as determined by the laboratories in which the blood was analyzed).

4.- T. cruzi antibodies titers mean reduction below the threshold used for diagnosis.

5.- Decrease of T. cruzi antibodies level greater than two titers.

If data are available, we will collect the outcome of interest every month for the first 2 years of follow-up after treatment. Subsequently, data will be collected annually.

### Secondary outcomes

None

## Data extraction, (selection and coding)

Collection of study-level data

Two reviewers will independently extract and record data from each eligible and included study. A pre-designed general data extraction form will be used after pilot testing. Disagreements will be resolved by discussion and, when necessary, a third reviewer will be consulted.

We will include the following general information:

-Source of study report: publication type, year of publication, journal, authors’ names, and language.

-Study location: geographical region, country, province, city, setting (urban vs. rural).

-Study population: sample size, age at enrollment, living in endemic area under surveillance or not under surveillance, visit to endemic area (even for a brief period), and dates of initiation and ending of data collection.

-Disease: definition of chronic T. cruzi infection, diagnostic tests (number and type of laboratory tests used), quality control measures.

-Trypanocidal treatment: drug, dose, age at administration and duration.

-Follow-up: duration (months), diagnostic tests (number and type of laboratory tests used), quality control, clinical assessment, comparison group.

Collection of individual participant data

One of the labor-intensive tasks in IPD meta-analysis is to obtain the original primary data of individual subjects. The fact that Dr. Sergio Sosa-Estani, Senior Researcher and leader of the review team, already belongs to collaborative research groups both at a regional and international level may facilitate this process.

We will first contact primary investigators to invite them to participate in the IPD MA by sending an email including:

a) a summary of our systematic review with a link to its protocol in PROSPERO,

b) the study(ies) of interest,

c) a brief online survey to track participation,

e) an agreement of data ownership and co-authorship,

d) a statement about confidentiality of individual participant data,

e) a request for their commitment to help with data management.

If researchers agree to collaborate with our research, then we will contact them to clarify any doubts or queries regarding our project and to provide additional information about how to send their study dataset. We will ask the authors of primary studies to supply a unique identification number for each participant. Furthermore, data will be sent in electronic format by encrypted e-mail, wherever possible.

We will accept raw databases in all formats, but we will develop a template with the preliminary list of variables of interest in an ad-hoc basis to ensure correct coding and merging of study data sets.

## Risk of bias (quality) assessment
Two review authors will independently assess quality of each included study using a pre-designed data extraction form with the principal domains of bias. The risk of bias in RCTs will be based on the Risk of Bias tool from Cochrane Collaboration. The risk of bias in observational studies will be based on the guidelines developed by Hayden et al. We will provide details for each study in a 'Risk of bias' table.

## Strategy for data synthesis
We have different possible meta-analytical scenarios. First, if we are interested in the occurrence of an event as reported by investigators of primary studies, a traditional aggregate meta-analysis is sufficient. Second, if we are interested in both the occurrence of an event and the time to event, then the best approach is a meta-analysis of individual participant data (IPD). Last, an approach combining aggregate and IPD meta-analysis may be considered (if suitable).

1) Meta-analysis using aggregate data obtained from published reports

According to the information reported, we will combine survival curves at each time of follow-up by using weighted averages. The weighting will be based on quality assessment, number of participants and rate of loss to follow-up of each available study.

We will also consider the grade of heterogeneity among included studies at each time of follow-up through I2 statistics.

To address the second objective, we will conduct a meta-analysis of Hazard Ratios (HRs). The HRs will give the overall relative chance of an event between two study groups.

In the first stage, we will estimate HRs for each trial by using the information reported and, in the second stage, we will pool these HRs.

2) Meta-analysis using IPD obtained from original study data sets

As high heterogeneity among included studies is expected, we will consider two strategies to conduct time to event analysis:

a.) To apply survival analysis methods like the log rank test for simple comparison and Cox regression for analyses when one or more variables are continuous. We will use a random effect model because it is more conservative by creating a wider CI around a pooled HR than the fixed effect analysis model.

b.) To apply a two-stage approach where we will create summary statistics out of the IPD in each study separately (stage 1) and combine the summary data obtained using a standard meta-analysis method (stage 2). We plan to use a standard Stats Package to obtain estimates of treatment effect and standard error (e.g. SAS or STATA) for stage 1, and then we will input data using Generic Inverse Variance Method in Rev Man [25] for stage 2. Another option will be to get free access to a specific software for IPD analysis.

Both strategies will allow us to estimate survival curves and HRs comparing two groups and/or subgroups of subjects.

We will use 95% confidence intervals (CI) for both the results of individual studies and the pooled estimates.

3) Combined meta-analysis using published summary study data and IPD

Following statistical advice, we might pursue a combined meta-analysis of aggregate data of studies for which IPD is not available with analyzed IPD.

### Analysis of subgroups or subsets

We will conduct the following subgroup analyses:

-Age of participants at time of treatment: pediatric/adolescent (less than 19 years old) vs. adult population.

-Type of serological test: conventional serology vs. non-conventional serology.

-Time elapsed between treatment and testing: early chronic phase (less than ten years) vs. late chronic phase (equal or more than ten years).

-Duration of follow-up: short-term follow-up (less than five years) vs. long-term follow-up (equal or more than five years).

-Region where the patient was infected: Central vs. South America. It is expected to find an earlier and higher rate of seroconversion in Central America due to the presence of different parasite lineages, i.e., T. cruzi type I predominating in Central America and T. cruzi type Non I (II, V and VI) in South America.

### Contact details for further information

Sergio Sosa-Estani

Instituto Nacional de Parasitología "Dr. Mario Fatala Chaben"

ANLIS Dr. Carlos G. Malbran,

Ministerio de Salud de la Nación

Av. Paseo Colón 568

1063 Ciudad de Buenos Aires, Argentina

ssosa@msal.gov.ar

### Organisational affiliation of the review

Instituto Nacional de Parasitología "Dr. Mario Fatala Chaben" ANLIS Dr. Carlos G. Malbran, Ministerio de Salud de la Nación

fatala@anlis.gov.ar

### Review team

Dr Yanina Sguassero, INP-ANLIS, Buenos Aires, Argentina. CREP, Rosario, Argentina.
Mrs Christine Christensen,
Dr Cristina Cuesta, Facultad de Ciencias Económicas y Estadística, UNR, Rosario, Argentina. CREP, Rosario, Argentina.
Ms Karen Roberts, Facultad de Ciencias Económicas y Estadística, UNR, Rosario, Argentina.
Mr Daniel Comandé, IECS, Buenos Aires, Argentina.
Dr Agustín Ciapponi, IECS, Buenos Aires, Argentina.
Mrs Daniel Giordano, CREP, Rosario, Argentina.
Ms Elizabeth Hicks, Duke University, NC, USA.
Dr Sergio Sosa-Estani, INP-ANLIS, Buenos Aires, Argentina.

### Collaborators

Professor Katrina Williams, Cochrane Prognosis Method Group, Australia
Dr Sue Woolfenden, Cochrane Prognosis Method Group, Australia

### Anticipated or actual start date

01 April 2013

### Anticipated completion date

15 December 2015

### Funding sources/sponsors

Savant, Inc. USA

Special Programme for Research and Training in Tropical Diseases (TDR), World Health Organization

### Conflicts of interest

This research was partially sponsored by Savant. Inc. However, the authors have no affiliation to this company in relation to employment, consultancy, patent of new products, etc.

Dr. Sergio Sosa-Estani is the principal investigator of two studies included in the systematic review.

### Language

English

### Country

Argentina

### Subject index terms status

Subject indexing assigned by CRD

### Subject index terms
Chronic Disease; Humans; Parasitology; Prognosis; Serologic Tests; Trypanosoma cruzi

### Stage of review
Completed but not published

### Date of registration in PROSPERO
19 March 2012

### Date of publication of this revision
15 May 2015


### DOI
10.15124/CRD42012002162


| Stage of review at time of this submission | Started | Completed |
| --- | --- | --- |
| Preliminary searches | Yes | Yes |
| Piloting of the study selection process | Yes | Yes |
| Formal screening of search results against eligibility criteria | Yes | Yes |
| Data extraction | Yes | Yes |
| Risk of bias (quality) assessment | Yes | Yes |
| Data analysis | Yes | Yes |

**PROSPERO**
**International prospective register of systematic reviews**
The information in this record has been provided by the named contact for this review. CRD has accepted this information in good faith and registered the review in PROSPERO. CRD bears no responsibility or liability for the content of this registration record, any associated files or external websites.
