# Supplementary material for: Course of Chronic Trypanosoma cruzi Infection after Treatment Based on Parasitological and Serological Tests: A Systematic Review of Follow-Up Studies
Source: PLoS One. 2015 Oct 5;10(10):e0139363. doi: 10.1371/journal.pone.0139363 (PMC4593559; doi:10.1371/journal.pone.0139363)
Supplement: S1 Table — Ag = antigen, AT-ELISA = chemiluminescent enzyme-linked immunosorbent assay with a trypomastigote mucin antigen, BZN = benznidazole, CF = complement fixation, CoML = complement mediated lysis, d = day, DA-2ME = direct agglutination with 2-mercaptoethanol, ELISA = enzyme-linked immunosorbent assay, ELISA-F29 = ELISA test using the F29 protein of T. cruzi, FC-ALTA-IgG = anti-live-trypomastigote antibodies, HA = hemagglutination assay, HC = hemoculture, IIF = indirect immunofluorescence assay, IF = immunofluorescence, IHA = indirect hemagglutination assay, IRA-ELISA = ELISA with an individual recombinant antigens, kg = kilogram, mg = milligram, mo = months, multiplex serological assay = a panel of 14 recombinant T. cruzi proteins in a Luminex-based format, NFTX = nifurtimox, PCR = polymerase chain reaction, rCRP = recombinant complement regulatory protein, RCT = randomised controlled trial, rt-PCR = real time PCR, RAM-ELISA = ELISA with a recombinant antigen mixture, SAPA = shed acute-phase antigen, TESA-blot = immunoblot assay, XD = xenodiagnosis, y = years. 1conventional “in house” serological tests (unless indicated otherwise). 2commercial conventional test. 3 non-conventional test. (PDF) [file pone.0139363.s009.pdf]

Source	Country	Study design	Age at treatment	Study groups	Duration of follow-up	Parasitological endpoints	Serological endpoints[1]
Aguiar et al., 2012 [33]	Brazil	Cohort	Adults	BNZ (n=29):<br>5 mg/kg/d for 60 days<br><br>Control (n=10):<br>no treatment	1-29 y	HC, Nested PCR, XD	IIF[2], ELISA[2]
Britto et al., 2001 [58]	Brazil	Cohort	Children & adults	BNZ (n=26):<br>5-6 mg/kg/d for 30-60 days<br><br>NFTX (n=27):<br>7-8 mg/kg/d for 60-90 days<br><br>Placebo (n=15)	20 y (mean)	PCR, XD	Not reported
Silveira et al., 2000 [62]			Children	BNZ (n=10):<br>5-7 mg/kg/d for 60 days<br><br>NFTX (n=2):<br>7-8 mg/kg/d for 60-90 days	8 & 20 y		ELISA, IHA, IIF
Cancado et al., 1976 [72]	Brazil	Cohort	Children & adults	NFTX (n=15):<br>10 mg/kg/d for 60-120 days<br><br>Placebo (n=5)	2 y	XD	CF, HA, IF
Cancado, 2002 [57]	Brazil	Cohort	Children & adults	BNZ (n=113):<br>5-10 mg/kg/d for 40 days	6-18 y	Not reported	CF, HA, IF, ELISA
Cerisola et al., 1977 [71]	Argentina<br>Brazil<br>Chile	Cohort	Adults	NFTX (n=109):<br>8-10 mg/kg/d for 60-120 days<br><br>Control (n=30):<br>no treatment	11 mo	XD	Not reported
Coura et al., 1997 [26]	Brazil	RCT	Adults	BNZ (n=26):<br>5 mg/kg/d for 30 days<br><br>NFTX (n=27):<br>5 mg/kg/d for 30 days<br><br>Placebo (n=24)	12 mo	XD	CF, IF
de Andrade et al., 1996 [27]	Brazil	RCT	Children	BNZ (n=64):<br>7.5 mg/kg/d for 60 days<br><br>Placebo (n=65)	3 y	Not reported	AT-ELISA[3], ELISA, IHA, IIF
Galvão et al., 2003 [24]				BNZ (n=58)<br><br>Placebo (n=53)		PCR	Not reported
Andrade et al., 2004 [23]					6 y	Not reported	AT-ELISA[3]
de Castro et al., 2006 [48]	Brazil	Cohort	Adults	BNZ (n=27):<br>5 mg/kg/d for 60 days<br><br>Control (n=13):<br>no treatment	24 mo	HC	Not reported
de Lana et al., 2009 [40]	Brazil	Cohort	Children & adults	BNZ (n=28):<br>5-10 mg/kg/d for 40-60 days	9 y	HC, PCR	ELISA,<br>FC-ALTA-IgG ELISA[3]
Machado-de-Assis et al., 2013 [30]					13 y		ELISA[2], IHA[2]

de Oliveira, 1990 [68]	Brazil	Cohort	Children & adults	BNZ (n=50):<br>5-8 mg/kg/d for 60 days<br><br>NFTX (n=50):<br>10-15 mg/kg/d for 60-120 days	5 to 18 y	XD	CF, IF, HA
Diniz-Marques et al., 2003 [56]	Brazil	Cohort	Children	BNZ (n=48):<br>5-10 mg/kg/d for 60 days	24 mo	Not reported	ELISA, IHA, IIF
Escribà et al., 2009 [41]	Honduras	Cohort	Children	BNZ (n=2,275):<br>5-7.5 mg/kg/d for 60days	18-36 mo	Not reported	ELISA[2]
Yun et al., 2009 [45]	Bolivia<br>Guatemala				18-60 mo &<br>36 mo		
Fabbro et al., 2000 [60]	Argentina	Cohort	Children & adults	BNZ (n=36):<br>5 mg/kg/d for 30 days<br><br>NFTX (n=32):<br>5-8 mg/kg/d for 60 days<br><br>Control (n=130):<br>no treatment	14 y (mean)	XD	DA-2ME, IHA, IIF
Fabbro et al., 2007 [47]			Adults	BNZ (n=27)<br><br>NFTX (n=27):<br>8-10 mg/kg/d for 45-60 days<br><br>Control (n=57)	21 y (mean)		DA-2ME, ELISA[2], IHA, IIF
Fabbro et al., 2010 [37]					23 y (mean)		
Fabbro et al., 2013 [28]				BNZ (n=13)<br><br>NFTX (n=16)<br><br>Control (n=37)		Not reported	DA-2ME, ELISA[2], IHA, IIF, ELISA-F29[3]
Fernandes et al., 2009 [42]	Brazil	Cohort	Children & adults	BNZ (n=80):<br>5 mg/kg/d for 60 days<br><br>Control (n=50):<br>no Chagas disease	36 mo	HC, PCR	ELISA[2], IIF[2]
Flores-Chavez et al., 2006 [49]	Bolivia	Cohort	Children	BNZ (n=35):<br>8 mg/kg/d for 60 days	12 mo	PCR, XD	ELISA, ELISA for SAPA[3]
Galvão et al., 1993 [67]	Brazil	Cohort	Adults	BNZ: 8-10 mg/kg/d for 30-60 days &NFTX: 5-7 mg/kg/d for 30-60 days (n=85)<br><br>Control (n=101): no treatment	3-10 y	HC	CF, CoML, IIF
Gallerano et al., 2000 [61]	Argentina	Cohort	Adults	BNZ (n=130):<br>4-8 mg/kg/d for 45-60 days<br><br>NFTX (n=96):<br>10 mg/kg/d for 45-60 days<br><br>Control (n=668):<br>no treatment	5,3 y	XD	IHA, IIF
Hasslocher-Moreno, 2010 [38]	Brazil	Cohort	Adults	BNZ (n=62):<br>5 mg/kg/for 30-60 days<br><br>Control (n=62):<br>no treatment	140,9 mo<br>(± 47.8)	XD	IIF[2]
Jackson et al., 2013 [29]	Switzerland	Cohort	Adults	NFTX (n=37):<br>10 mg/kg/d for 30-60 days	3 y	PCR	ELISA

Lacunza et al., 2006 [50]	Argentina	Cohort	Adults	BNZ (n=14):<br>5 mg/kg/d for 60 days<br><br>Control (n=5):<br>no treatment	6 mo	PCR	ELISA[2], IHA[2]
Levi et al., 1971 [74]	Brazil	Cohort	Adults	NFTX (n=12):<br>5-17 mg/kg/d for 120 days	3 y	Not reported	CF
Machado-de-Assis et al., 2012 [34]	Brazil	Cohort	Children & adults	BNZ (n=94):<br>5 mg/kg/d for 60 days	16.9 y<br>(± 6.8)	HC, PCR	ELISA[2], IHA[2], IIF[2], TESA-blot
Maekelt, 1969 [75]	Venezuela	Cohort	Adults	NFTX (n=14):<br>5-15 mg/kg/d for 120 days	10 mo	Not reported	CF, IHA
Maldonado et al., 1995 [65]	Paraguay	Cohort	Children	BNZ (n=20):<br>5-7 mg/kg/d for 60 days	6 mo	HC, PCR, XD	ELISA, IIF
Vera de Bilbao et al., 2004 [54]				BNZ (n=12)	24 mo	XD	
Meira et al., 2004 [53]	Brazil	Cohort	Adults	BNZ (n=31):<br>5 mg/kg/d for 60 days	27.7 mo<br>(mean)	HC, PCR	CoML, ELISA, IHA, IIF, rCRP ELISA[3]
Molina et al., 2014 [22]	Spain	RCT	Adults	BNZ (n=26):<br>150 mg twice daily for 60 days	10 mo	rt-PCR	ELISA[2]
Moretti et al., 1998 [63]	Argentina	Cohort	Children & adults	BNZ & NFTX (n=42). No further details were provided.<br><br>Control (n=42):<br>no treatment	2-20 y	Not reported	HA, IF, ELISA[3]
Muñoz et al., 2013 [31]	Chile	Cohort	Adults	NFTX (n=21):<br>6 mg/kg/d for 60 days	13 mo	rt-PCR, XD	Not reported
Murcia et al., 2010 [39]	Spain	Cohort	Children & adults	BNZ (n=181):<br>5-7 mg/kg/d for 60 days	35 mo	PCR	IIF[2]
Neves da silva et al., 1974 [73]	Brazil	Cohort	Adults	NFTX (n=15):<br>8-10 mg/kg/d for 120 days<br><br>Placebo (n=10)	48 mo	XD	HA, IF
Pérez Ayala et al., 2011 [35]	Spain	Cohort	Adults	BNZ (n=64):<br>5 mg/kg/for 30-60 days	1 y<br>(mean)	PCR	ELISA, IHA
Rumi et al., 2013 [32]	Argentina	Cohort	Children	BNZ (n=221):<br>5 mg/kg/d for 60 days	2-5 y	PCR	ELISA[2], IHA[2]
Sánchez Negrette et al., 2008 [46]	Argentina	Cohort	Adults	BNZ (n=18):<br>5 mg/kg/d for 60 days	24-66 mo	Not reported	CMA-ELISA[2], IRA-ELISA & RAM-ELISA[2,3], IHA[2]
Shenone et al., 1981 [70]	Chile	Cohort	Adults	BNZ (n=6):<br>8-15 mg/kg/d for 30 days<br><br>NFTX (n=15):<br>5-15 mg/kg/d for 60-120 days<br><br>Placebo (n=8) for 120 days	83 mo (mean)	XD	Not reported
Solari et al., 1998 [64]	Chile	Cohort	Children	NFTX (n=28):<br>7 mg/kg/d for 60 days	6 mo	PCR, XD	Not reported
Solari et al., 2001 [59]				NFTX (n=66)	3 y		ELISA, HAI

Streiger et al., 2004 [55]	Argentina	Cohort	Children	BNZ (n=64):<br>5 mg/kg/for 30 days<br><br>NFTX (n=7):<br>12-15 mg/kg/d for 45-60 days<br><br>Control (n=24):<br>no treatment	12 y (median)	Not reported	DA-2ME, ELISA[2], IHA, IIF
Sosa-Estani et al., 1998 [25]	Argentina	RCT	Children	BNZ (n=55):<br>5 mg/kg/d for 60 days<br><br>Placebo (n=51)	4 y	XD	ELISA,<br>ELISA F29[3],<br>IHA, IIF
Sosa-Estani et al., 2009 [43]		Cohort	Adults	BNZ (n=16)	14 y	PCR, XD	
Vera de Bilbao et al., 2006 [51]	Paraguay	Cohort	Children	BNZ (n=5):<br>5-7 mg/kg/d for 60 days	10 y	Micromethod	ELISA, IIF
Vigliano et al., 1989 [69]	Argentina	Cohort	Adults	BNZ (n=131):<br>5 mg/kg/d for 35 days<br><br>Control (n=70):<br>no treatment	7.5 y (mean)	Not reported	CF, IHA, IIF
Viotti et al., 1994 [66]	Argentina	Cohort	Adults	BNZ (n=131):<br>5 mg/kg/d for 30 days<br><br>Control (n=70):<br>no treatment	8 y (mean)	XD	CF, IHA, IF
Viotti et al., 2006 [52]	Argentina	Cohort	Adults	BNZ (n=283):<br>5 mg/kg/d for 30 days<br><br>Control (n=283):<br>no treatment	9.8 y (mean)	Not reported	CF, IHA, IIF
Viotti et al., 2009 [44]	Argentina	Cohort	Adults	BNZ (n=760):<br>5 mg/kg/d for 30 days<br><br>Control (n=1.075):<br>no treatment	10.4 y<br>(± 7.8)	Not reported	ELISA, IHA or IIF
Viotti et al., 2011 [36]	Argentina	Cohort	Adults	BNZ (n=53):<br>5 mg/kg/d for 30 days<br><br>Control (n=89):<br>no treatment	36 mo (mean)	Not reported	ELISA, IHA,IIF, multiplex serologic assay[3]
