# Supplementary material for: Course of Chronic Trypanosoma cruzi Infection after Treatment Based on Parasitological and Serological Tests: A Systematic Review of Follow-Up Studies
Source: PLoS One. 2015 Oct 5;10(10):e0139363. doi: 10.1371/journal.pone.0139363 (PMC4593559; doi:10.1371/journal.pone.0139363)
Supplement: S2 Table — BNZ = benznidazole, d = day, kg = kilogram, mg = milligram, NFTX = nifurtimox. (PDF) [file pone.0139363.s010.pdf]

| **Source** | **Country** | **Study groups** | **Reasons for exclusion** |
|---|---|---|---|
| *Libonatti et al., 1979* [83] | Argentina | NFTX (n=4,441): 8 mg/kg/d for 90 days up to 1976 and for 60 days onwards | Monograph with lack of key numerical data about follow-up of subjects |
| *Apt et al., 1986* [82] | Chile | A) BNZ (n=5): doses starting at 2 mg/kg increasing to 5 mg/kg/d by the fifth day<br><br>B) BNZ plus obioactin (n=8) for 35 days<br><br>C) Control (placebo) (n=8) | No parasitological and/or serological outcomes were reported |
| *Carpintero, 1993* [81] | Argentina | BNZ (n=2,937): 5 mg/kg/d plus tioctic acid: 20 mg/d plus magnesium lactate: 1 mg plus pyridoxine: 250 mg for 30 days | Combined trypanocidal treatment |
| *Guevara et al., 1994* [80] | Ecuador | BNZ (n=2): 3 mg/kg for 60 days | Short report on the use of a simple ELISA utilizing a recombinant *T. cruzi* protein (rTc24) as antigen to monitor cure of patients with acute Chagas disease |
| *Neto, 1998* [79] | Brazil | | Short viewpoint of the author about treatment for Chronic *T. cruzi* infection |
| *Lauria-Pires et al., 2000* [78] | Brazil | BNZ (n=17): 10 mg/kg/d for 30-60 days<br><br>NFTX (n=28):10 mg/kg/d for 30-60 days for 20, 30 and 60 days<br><br>Control (n=46): no treatment | Case-control study |
| *Fernández-Villegas et al., 2001* [76] | Spain | BNZ (n=46): 5 mg/kg/d for 60 days | Only results of non-conventional serological tests were performed and reported |
| *Shenone et al., 2003* [77] | Chile | NFTX: 7-10 mg/kg/d for 30 days | Letter to the editor |
